# 2-phenylacetamide Separated from the seed of *Lepidium apetalum* Willd. inhibited renal fibrosis via MAPK pathway mediated RAAS and oxidative stress in SHR Rats

**DOI:** 10.1186/s12906-023-04012-w

**Published:** 2023-06-23

**Authors:** Pei-pei Yuan, Meng Li, Qi Zhang, Meng-nan Zeng, Ying-ying Ke, Ya-xin Wei, Yang Fu, Xiao-ke Zheng, Wei-sheng Feng

**Affiliations:** 1 College of Pharmacy, Henan University of Chinese Medicine, Zhengzhou, 450046 China; 2 The Engineering and Technology Center for Chinese Medicine Development of Henan Province, 156 Jinshui East Road, Zhengzhou, 450046 China

**Keywords:** *Lepidium apetalum* Willd., 2-phenylacetamide, Hypertension, Renal fibrosis, RAAS, Oxidative stress, MAPK signalling pathway

## Abstract

**Background:**

Renal fibrosis with Renin–angiotensin–aldosterone system (RAAS) activation and oxidative stress are one of the major complications in hypertension. 2-phenylacetamide (PA), a major active component of *Lepidium apetalum* Willd. (L.A), has numerous pharmacological effects. Its analogues have the effect of anti-renal fibrosis and alleviating renal injury. This study aims to explore the underlying mechanism of PA for regulating the renal fibrosis in SHR based on the MAPK pathway mediated RAAS and oxidative stress.

**Methods:**

The SHR rats were used as the hypertension model, and the WKY rats were used as the control group. The blood pressure (BP), urine volume were detected every week. After PA treatment for 4 weeks, the levels of RAAS, inflammation and cytokines were measured by Enzyme-Linked Immunosorbnent Assay (ELISA). Hematoxylin–Eosin staining (HE), Masson and Immunohistochemistry (IHC) were used to observe the renal pathology, collagen deposition and fibrosis. Western blot was used to examine the MAPK pathway in renal. Finally, the SB203580 (p38 MAPK inhibitor) antagonism assay in the high NaCl-induced NRK52e cells was used, together with In-Cell Western (ICW), Flow Cytometry (FCM), High Content Screening (HCS) and ELISA to confirm the potential pharmacological mechanism.

**Results:**

PA reduced the BP, RAAS, inflammation and cytokines, promoted the urine, and relieved renal pathological injury and collagen deposition, repaired renal fibrosis, decreased the expression of NADPH Oxidase 4 (NOX4), transforming growth factor-β (TGF-β), SMAD3 and MAPK signaling pathway in SHR rats. Meanwhile,,the role of PA could be blocked by p38 antagonist SB203580 effectively in the high NaCl-induced NRK52e cells. Moreover, molecular docking indicated that PA occupied the ligand binding sites of p38 MAPK.

**Conclusion:**

PA inhibited renal fibrosis via MAPK signalling pathway mediated RAAS and oxidative stress in SHR Rats.

**Supplementary Information:**

The online version contains supplementary material available at 10.1186/s12906-023-04012-w.

## Introduction

Hypertension is a systemic disease characterized by elevated arterial pressure, which can be accompanied by functional or organic changes in the heart, kidneys and other organs. Currently, 1/3 of the people in the world suffer from hypertension [1]. It can cause complications in target organs such as heart, brain, kidney, eye and blood vessel, among which kidney is one of the most susceptible organs. According to National Guidelines for Prevention and Management of Basic Hypertension, 2020 edition, The number of hypertensive patients in China has reached 245 million. High disability and mortality caused by severe complications of hypertension, including kidney disease, stroke and coronary heart disease, have become a heavy burden on Chinese families and society. In some patients in China, hypertensive renal damage has replaced glomerular disease as the second cause of end-stage renal disease. However, high blood pressure can be prevented and controlled. Prevention and control of hypertension is one of the core strategies to curb the prevalence of complications such as kidney disease.

The pathogenesis of hypertension is complicated and has not been completely clear by far. RAAS is an important neurohumoral mechanism that regulates fluid metabolism, vasoconstriction and blood pressure stabilization. The active Ang II produced by the RAAS system can cause vasoconstriction, promote aldosterone synthesis and secretion, and increase circulating blood flow, thus leading to hypertension [2]. The kidney is the effector organ of Ang II. Meanwhile, renal fibrosis is the most common complication of renal injury caused by hypertension. Recent studies have shown that hypertension activates RAAS, increases Ang II level and endothelin-1 (ET-1) expression, stimulates vascular endothelial injury, increases ROS production and promotes oxidative stress [3–6]. At the same time, RAAS, and specifically Ang II, act as a key stimulator of NOX (such as NOX4 in the kidney), contributing to ROS-mediated damage in many pathologies including renal fibrosis with hypertension [7].

MAPKs are a class of intracellular serine/threonine protein kinases with multiple parallel signaling pathways, including p44/42, JNK and p38. As an important signaling molecule mediating cell function, MAPK plays a key role in the molecular mechanism of target organ damage in hypertension. Ang II and vascular endothelial growth factor can activate ERK and JNK in mesangial cells through G protein-coupled receptors. Inflammatory factors such as TNF-α and IL-1β activate multiple MAPK signaling pathways in renal cells depending on oxidative stress. On the contrary, some vasodilator factors such as prostaglandins and dopamine can inhibit the activity of ERK in renal cells, and heparin, cAMP and cGMP also have similar effects, which may be a protective effect. The above studies suggest that MAPK signaling pathway is closely related to target organ damage of hypertension under various stimulus factors, and is especially involved in the chronic progression of target organ damage.

The seed of *Lepidium apetalum* Willd. (L.A) was first recorded in Shennong Herbal Classic. It is a commonly used Chinese herbal medicine in the treatment of edema, cough and asthma, and contains a variety of secondary metabolites and widely biological activities [8, 9]. At present, the studies on constituents and pharmacological activities of L.A showed that it contains various types of secondary metabolites, such as oils, flavonoids, sterols and cardiac glycosides, and shows antioxidant, antibacterial and beneficial cardiac activities [10, 11]. In the early stage, the chemical composition of L.A was systematically separated by our research group. We isolated the main compounds from LA and obtained 2-phenylacetamide (PA) and some other compounds in the previous study [12]. The content of PA was the highest in L.A.

PA, as the major parent nucleus, is a key pharmaceutical intermediate of atenolol [13] and penicillin [14]. Studies have shown that atenolol have beneficial effects on renal function by improving glomerular filtration rate, effective renal plasma flow and total renal resistance[15]. Meanwhile, the neurotransmitter dopamine, which uses PA as its parent nucleus, could regulate fibronectin and collagen I to buffer renal injury [16]. These studies showed that similar structural compounds of PA could improve renal fibrosis in nephropathy, suggesting that PA, as the basic mother nucleus, may have a potential protective effect on renal injury. Meanwhile, PA has small molecular weight, easy to prepare and obtain, and has a strong druggability. Our previous studies in vivo have confirmed that PA showed antihypertensive effect, and also improve hypertension-induced myocardial damage [17]. In addition, PA analogue ***cis***-desulfoglucotropaeolin and ***trans***-desulfoglucotropaeolin may play a role in the treatment of kidney injury and other diseases (including nephritis, renal fibrosis and hypertension) [18]. As renal fibrosis is an important complication in the progression of hypertension, we consider whether PA could ameliorate renal fibrosis with hypertension. Therefore, the mechanism of anti-renal fibrosis effect of PA in hypertension was studied using the spontaneously hypertensive rat (SHR) and the high NaCl-induced NRK52e cell [18], combined with SB203580 antagonistic experiment.

## Materials and methods

### Material

*Lepidium apetalum* Willd. (L.A), obtained from Nanyang County, Henan Province, China, was authenticated by professors Dong Chengming of HUCM. We received approval for sampling in line with the regulations of he National Key Research and Development Project—The Major Project for Research of the Modernization of TCM (2019YFC1708802). The voucher specimens (No. 20210715A) were deposited in the Laboratory of Chinese Medicine Chemistry Laboratory in HUCM.

**Fig.1:**
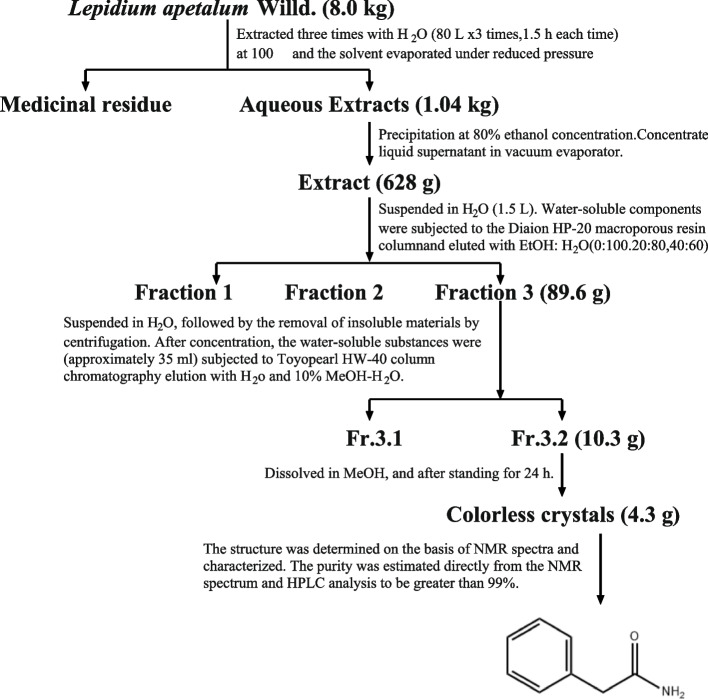
Separation and chemical structure of 2-phenylacetamide

PA was extracted by water, which was subsequently concentrated, and extracted with ethanol as described before, and separated by different columns and finally obtained from the seed of L.A. (Fig. 1). The chemical profile has previously been analyzed by ultra-high performance liquid chromatography. Through NMR and HPLC analysis, the purity was to be greater than 99% [19].

### Animals and experimental protocol

Forty male SHR rats and eight maleWKY rats (mean weight was 180–200 g, certificate No.11400700256939) were purchased from Charles River Laboratories (No. SCXK (Beijing) 2017–0001). The mice were housed in a standard 12 h light/dark cycle at 22 ± 2 °C with 55 ± 10% humidity. All of the animal procedures were performed in accordance with the Guidelines for Care and Use of Laboratory Animals of the Henan University of Chinese Medicine, and the experiments were approved by the Animal Ethics Committee of Henan University of Chinese Medicine (DWLL201903052). The study was carried out in compliance with the ARRIVE guidelines.

Forty SHR rats were randomly divided into the model group (provided with saline solvent), hydrochlorothiazide (HCTZ, Changzhou Pharmaceutical Factory Ltd., China) group (17 mg/kg, SHR + HCTZ), PA low-dose (15 mg/kg, SHR + 15), middle-dose (30 mg/kg, SHR + 30) and high-dose (45 mg/kg, SHR + 45) group according to body weights and blood pressure. Eight WKY rats were used as the control group (provided with saline solvent). After treatment for 3 weeks, all animals were fasted overnight, anesthetized by intraperitoneal injection of 1% pentobarbital sodium until the righting reflex disappeared. Blood samples from the aorta and the renal tissues in each group were collected for further investigation.

### Measurements of blood pressure and urine output

The rats were placed in a fully automatic non-invasive blood pressure meter for 3 days to adjust to the environment for 1 h per day and appropriate blocking pressure was given during the adaptation period. During the drug intervention, blood pressure was measured once a week. After the heart rate was stabilised, BP of each rat was tested three times and the average value was taken as the final measurement result.

The body weight of the rats was measured daily. Before and during the drug intervention, a rat was placed in a metabolic cage and urine collected 24 h after the gavage was collected once a week to calculate the change in urine volume.

### Histopathological examination of renal pathology

The fresh left renal tissues were quickly fixed in 3.7% paraformaldehyde for 24 h, cleared in xylene, embedded in paraffin, and cut into 4 mm thickness by using a microtome. The slices were put into xylene I for 20 min- xylene II for 20 min- anhydrous ethanol I for 5 min- anhydrous ethanol II for 5 min-75% alcohol for 5 min, and rinsed with tap water. Then, the pathological changes were observed by different staining and immunological detection with slices.

Hematoxylin–eosin (HE) staining: After the sections were stained with hematoxylin and eosin successively, they were dehydrated and sealed for microscopic examination and image collection and analysis.

Masson staining: The sections were stained with potassium dichromate, iron hematoxylin, Lichun red acid magenta, molybdate and aniline blue successively. After differentiation and transparent sealing, microscopic examination was carried out, and the images were collected and analyzed. 5 pictures were randomly taken from each group and analysed with IPP 6.0 software.

Immunohistochemistry: The sections were dewaxed to water, followed by antigen repair, blocking endogenous peroxidase, and serum sealing. DAB color development was performed by incubation of primary (NOX4, Proteintech, Wuhan, China) and secondary antibodies. Then the nucleus was restained, dehydrated and sealed, and finally observed by microscope. 5 pictures were randomly taken from each group and analysed with IPP 6.0 software.

Immunofluorescence: The sections were dewaxed to water, followed by antigen repair, painting circle, hydrogen peroxide sealing and serum sealing. After that, the first primary antibody and the corresponding HRP labeled secondary antibody were added for incubation successively, and then CY3-TSA (or 488-TSA) was added. After microwave treatment, the mixed reagents of the second and third primary antibodies and the corresponding secondary antibodies were added for incubation successively. Then, after restaining the nucleus with DAPI, the spontaneous fluorescence of the tissue was quenched and then sealed. Finally, the images were collected.

### ELISA measurement

The tissue was cleaned with pre-cooled PBS (0.01 M, pH = 7.4). After weighing, PBS was added at a weight ratio of 1:9, and thoroughly broken on ice. The solvent were centrifuged at 5,000 g for 5–10 min and collected the supernatant. The levels of Renin, ACE, Ang II, ALD, COX2, PGE2, IL-1β, ET-1, E-selectin, MCP-1, TGF-β1, FN in serum and renal in each group were detected by ELISA according to the ELISA kit instructions (Elabscience Biotechnology Co., Ltd., Wuhan, China).

### Measurements of oxidative damage indicators

The tissue homogenate was taken according to the method of ELISA measurement. The content of SOD in each group was detected by hydroxylamine method. The content of MDA was detected using the thiobarbituric acid (TBA) method (Nanjing Jiancheng Bioengineering Institute, Nanjing, China).

### Western blot

The cytoplasm and nuclear protein of each group were obtained by protein extraction kit and the concentration was measured by BCA method (Beijing Solarbio Science and Technology Co., Ltd., Beijing, China). Each group of protein was loaded at 40 μg, electrophoretically separated in a 10–12% SDS-PAGE gel and the protein sample in the gel was transferred to PVDF membrane by a semi-dry method (Bio-Rad, CA, USA). 5% skim milk powder or BSA was blocked for 2 h and the corresponding primary antibody was added to incubate for 0.5 h, overnight at 4 °C and incubated for 0.5 h (CST, MA, USA). After washing for 5 min × 5 times, added the corresponding secondary antibody and incubated for 1 h. P-p44/p42、p-p38、HRP-conjugated anti-rabbit secondary antibody (CST, MA, USA); p44/p42、p38 (CST, MA, USA); HSP27 (CST, MA, USA); p-JNK antibody (GeneTex, CA, USA); JNK antibody (Immunoway, TX, USA); GAPDH (ABclonal, Wuhan, China). After 5 min × 5 washes, the bound antibodies were detected by ECL chemiluminescence (Merck Millipore, Billerico, USA), protein bands were semiquantified using Image Lab analysis software. In order to detect proteins with different molecular weights in this experiment and save antibody reagents, the blots were cut prior to hybridisation with antibodies according to the Marker molecular weight indication. Three independent parallel experiments were performed for each protein to ensure the accuracy and reliability of the results.

### Cell culture and experimental protocol

Rat renal proximal tubular epithelial cell lines (NRK52e) were purchased from ATCC (Rockville, MD, USA). The cells were cultured in Dulbecco's modified Eagle's medium (Hyclone, UT, USA) supplemented with 5% FBS (Gibco, MA, USA) at 37℃ in the humidified incubator with 5% CO_2_.

NRK52e cells were divided into the following groups: the control group (Control), the high NaCl-induced model group (High-NaCl) (treated with 200 mmol/L NaCl for 6 h), the HCTZ group (High-NaCl + HCTZ) (treated with 200 mmol/L NaCl and 10 μmol/L HCTZ for 6 h), the different dose PA group (treated with 200 mmol/L NaCl and 1, 5, 10 μmol/L PA seperately for 6 h). At the same time, each group was set with corresponding SB203580 control group, and 10 µM SB203580 was added 1 h before modeling.

NRK52e cells were seeded in 96-well plates at the density of 2 × 10^4^ cells/mL. After the cells became adherent, we removed the original medium and washed the cells. The medium of the normal control group was replaced with DMEM containing 10% FBS. The medium of the model group was replaced with DMEM containing 10% FBS with 200 mmol/L NaCl. Other group replaced the medium with corresponding component. After the cells were cultured for 6 h, we detected the cell survival rate using MTT in each group [18].

### In-cell western

The cells were uniformly inoculated into a light-shielded 96-well plate and the drug intervention was performed when the cell volume of each well was 80–90%. After a certain period of time, the medium was discarded and the cells were fixed. For the non-phosphorylated protein to be tested, after incubation in 3.7% formaldehyde, the cell membrane was infiltrated with 0.1% Triton. For the phosphorylated protein to be tested, the cells were incubated in methanol for 10 min. After fixation, incubated for 1 h in 5% skim milk powder. The primary antibody was diluted with 5% skim milk powder, using the primary antibody dilution ratio described in the instructions and incubated at 4 °C for 16–24 h (CST, MA, USA). Unbound primary antibody was removed by washing with PBST. Fluorescent-labelled secondary antibody (LI-COR, NE, USA) diluted with 5% skim milk powder was added at a dilution ratio of 1:1,000–1:5,000, incubated for 1 h, unbound secondary antibodies were washed and removed, the liquid in the well was drained and the cells were kept moist. The Odyssey two-colour infrared fluorescence imaging system (LI-COR, NE, USA) was used for the detection and the data was quantified using Image Studio Ver 5.2 software.

### Flow Cytometry (FCM)

At the end of the experiment, cell suspension of each group was collected, centrifuged at 1000 rpm for 5 min, and the precipitation was obtained. DCFH-DA diluted with 100 μL PBS was added and placed in the cell incubator with 37 °C for 20 min in darkness. Then, the samples were centrifuged at 1000 rpm for 5 min and the supernatant was discarded. PBS was added to the suspended cells, centrifuged at 1000 rpm for 5 min, repeated 3 times, and unbound probes were washed away. ROS content was determined by FlowSight multidimensional panoramic flow cytometer (Merck Millipore, Billerico, USA). The results were analysed with IDEAS 6.2.

### High Content Screening (HCS)

NRK52e cells were uniformly inoculated at a density of 5 × 10^4^/mL in 96-well plates (PerkinElmer, MA, USA). The drug intervention was performed when the cell volume of each well was 80–90%. After the experiment, the cells were washed with PBS, fixed with 4% paraformaldehyde for 20 min, then infiltrated with 0.1% Triton for 20 min. The treated cells were blocked with 5% goat serum at room temperature for 1 h and incubated with different primary antibodies at 4℃ overnight. Then incubated with secondary antibodies: Cy3 Goat Anti-Rabbit IgG (Abclonal, Wuhan, China) or FITC Goat Anti-Mouse IgG (Abclonal, Wuhan, China) for 1 h at room temperature. Finally, they were stained with DAPI for 10 min at room temperature after washing with PBS. High-content imaging system (PerkinElmer, MA, USA) was used to scan and obtain the images. The relative protein expression level was normalized using Harmony 4.8.

### Molecular docking

The structural formula of PA were obtained from PubChem database (https://pubchem.ncbi.nlm.nih.gov/). We downloaded and imported the p38 MAPK crystal complex structure (PDB ID: 4EYJ) from the Protein Data Bank (PDB) library, and performed molecular docking after preprocessing. Ten import the ligand molecules (PA) and receptor molecules (p38 MAPK) into Discovery Studio 2020 Client. Use Dock Ligands (CDOCKER) for semi-flexible docking. The process was performed as described in the literature [20].

### Data processing analysis

SPSS 20.0 (IBM, New York, NY, USA) was used to perform the data analysis. For quantitative data, one-way ANOVA was followed by t-test, and Dunnett’s test or Tukey’s test was performed according to the homogeneity of variance. All data are presented as mean ± SD. *P* < 0.05 was accepted as significant. The graphs were created by GraphPad Prism software (version 6, GraphPad Software, Inc., San Diego, CA, USA).

## Results

### PA improved the blood pressure, urine output and ranal histopathology of SHR

As shown in Table 1, the blood pressure of the SHR was raised, the urine volume was decreased significantly (*P* < 0.05). The administration of PA mitigated the blood pressure and augmented the urine volume significantly (*P* < 0.01 or *P* < 0.05), indicated that PA showed the effect of anti-hypertensive and promoting urination. Meanwhile, with the increase of dose, 45 mg/kg PA showed better improvement.

**Table 1:** Effect of PA on blood pressure and urine volume in SHR rats

Groups	BP (mmHg)			Urine Output (mL)
	SBP	MAP	DBP	
WKY	81.72 ± 23.50	72.65 ± 16.15	67.10 ± 10.87	7.36 ± 1.97
SHR	176.21 ± 17.03^**^	138.67 ± 33.06^**^	121.72 ± 15.03^**^	1.79 ± 0.87^**^
SHR + HCTZ	103.43 ± 23.26^##^	94.57 ± 17.62^##^	90.49 ± 18.42^##^	4.16 ± 0.98^##^
SHR + 15	142.54 ± 32.17^#^	119.93 ± 29.35	113.51 ± 27.25	2.20 ± 1.06
SHR + 30	129.77 ± 20.73^##^	111.90 ± 23.59^#^	99.71 ± 15.34^#^	4.63 ± 1.82^##^
SHR + 45	123.18 ± 31.71^##^	106.85 ± 25.46^##^	92.88 ± 21.15^##^	6.52 ± 1.53^##^

Data are expressed as mean ± SD, *n* = 8 per group. ^*^P < 0.05, ^**^P < 0.01 vs. WKY group; ^#^*P* < 0.05, ^##^*P* < 0.01 vs. SHR group

**Fig. 2:**
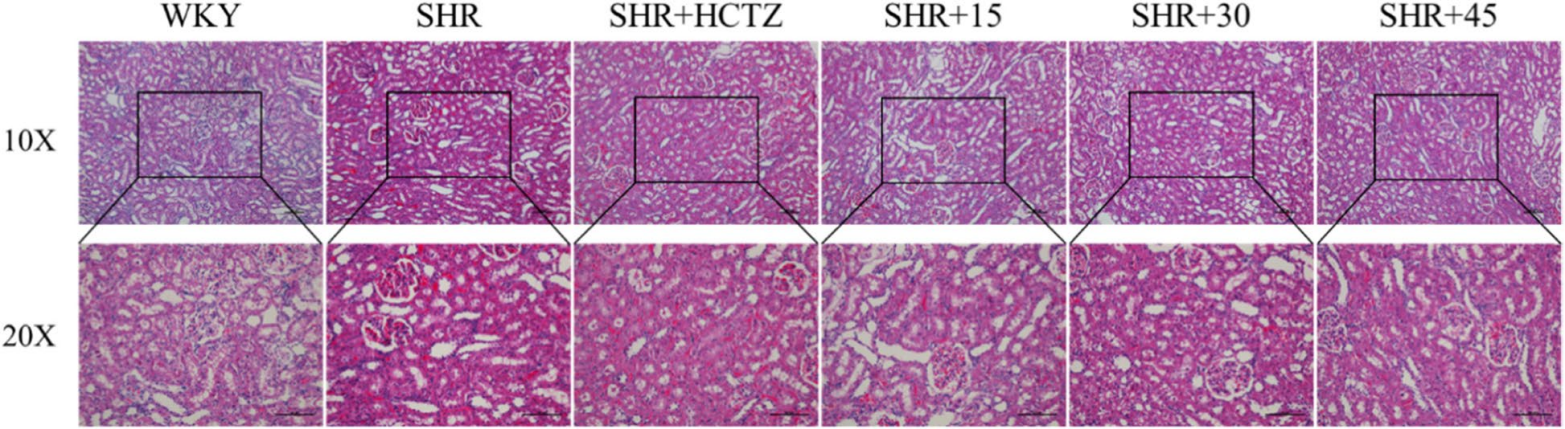
Effect of 2-phenylacetamide on renal pathological structure changes (HE staining,100 × and 200 ×)

HE staining showed that the WKY renal tissue morphology was normal, the arrangement of its epithelial cells was neat, no obvious fibrosis changes were found in the renal stroma; Renal tissue of SHR group showed significant atrophy, the arrangement of epithelial cells was not neat, its visible lymphocytes, neutrophils and monocytes infiltration, forming infiltrating fluid surrounding the glomeruli, inflammatory infiltration was significantly enhanced compared with WKY rats. After PA intervention, the above morphological changes were alleviated and the inflammatory infiltration of renal was improved to varying degrees (Fig. 2).

### PA relieved CVF and the expression of TGF-β and Smad3 of renal in SHR

**Fig. 3:**
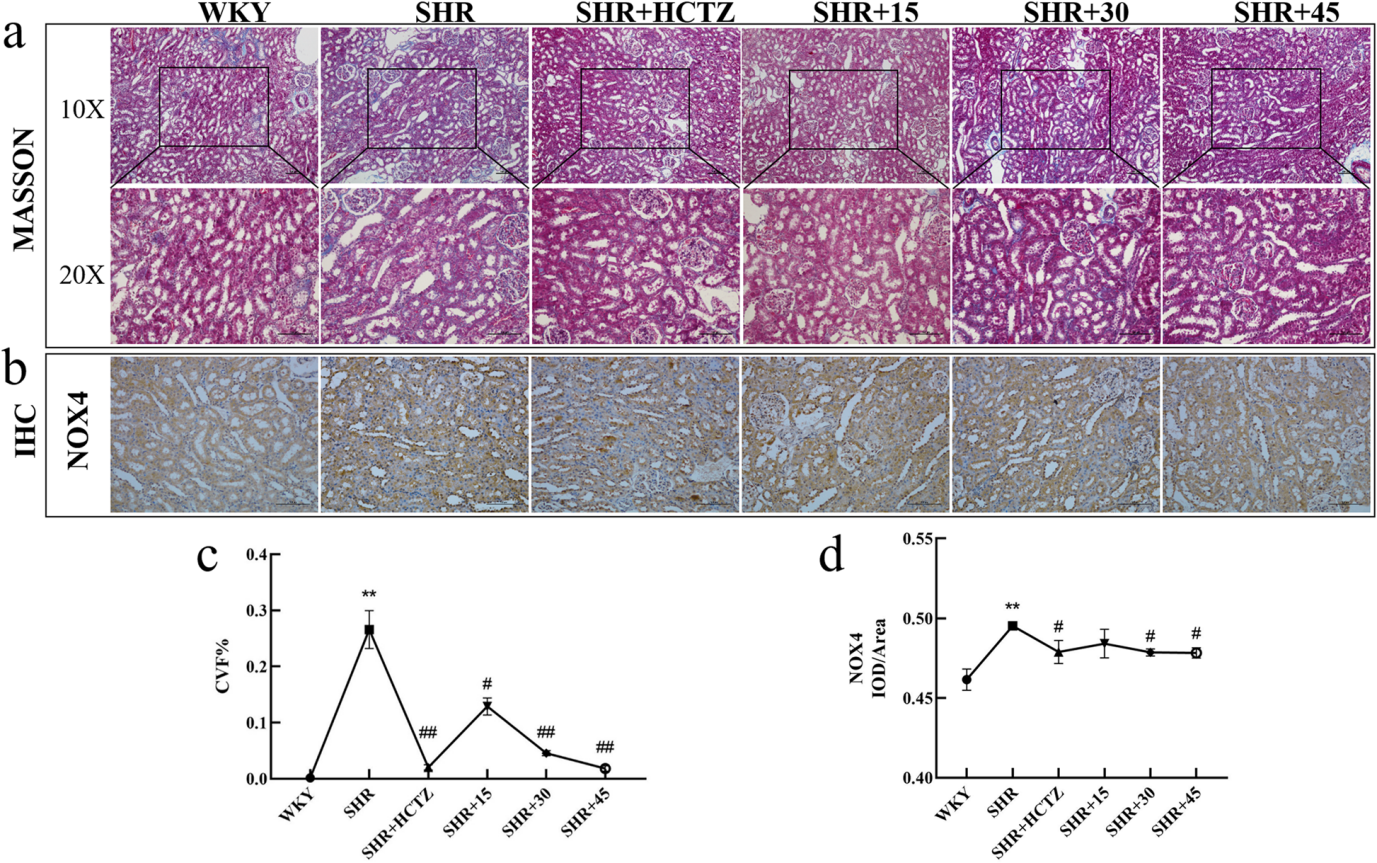
Effect of 2-phenylacetamide on CVF and the expression of NOX4 of renal in SHR. **a** MASSON staining, 100 × and 200 × . **b** The NOX4 expression with Immunohistochemistry. **c** The percentage of collagen volume fraction with MASSON. **d** The relative expression level of NOX4 with IHC. *n* = 5–8. ^*^ P < 0.05 or ^**^ P < 0.01 vs. WKY group; ^#^ *P* < 0.05 or ^##^ *P* < 0.01 vs. SHR group

**Fig. 4:**
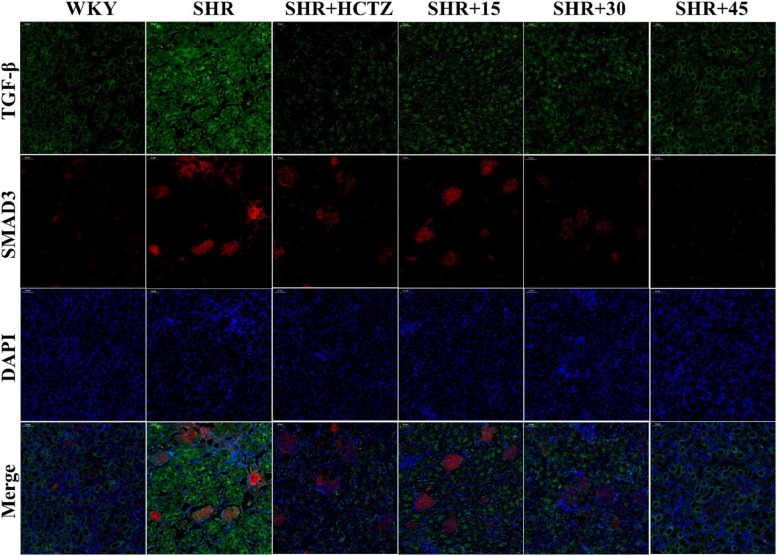
Effect of 2-phenylacetamide on the expression of TGFβ and Smad3 of renal in SHR. Immunofluorescence was used to detect the expression of TGFβ and Smad3 (200 ×)

The Masson staining results of renal tissue showed that interstitial fibrosis appeared locally in SHR group of renal tissue, along with obvious collagen deposition and the CVF was increased significantly (Fig. 3a, c). Immunofluorescence results indicated the increasing of TGF-β and Smad3 in SHR group (Fig. 4). The above results suggest that fibrosis collagen deposition occured in renal of SHR rats. As shown in Table 2, the levels of TGF-β1 and fibronectin (FN) in renal in SHR were significantly augmented, indicating that the degree of fibrosis was aggravated.

**Table 2:** Effect of PA on the content of fibrosis indicators in renal of SHR rats

Groups	TGF-β (pg/mL)	FN (pg/mL)
WKY	1548.55 ± 177.22	275.76 ± 34.74
SHR	3705.17 ± 516.48^**^	480.41 ± 33.72^**^
SHR + HCTZ	3244.71 ± 51.03	400.04 ± 25.42
SHR + 15	2750.08 ± 337.07^##^	440.75 ± 19.25
SHR + 30	2629.00 ± 511.91^##^	417.86 ± 71.80
SHR + 45	2549.39 ± 300.90^##^	407.58 ± 51.70^#^

Data are expressed as mean ± SD, *n* = 8 per group. ^*^ P < 0.05, ^**^ P < 0.01 vs. WKY group; ^#^* P* < 0.05, ^##^* P* < 0.01 vs. SHR group

After PA intervention, collagen deposition and the expression of TGF-β and Smad3 were significantly reduced (*P* < 0.01 or *P* < 0.05), and the levels of TGF-β1 and FN in each group were significantly diminished (*P* < 0.01 or *P* < 0.05). Results showed that PA mitigated the renal fibrosis state and restrainted the levels of cell chemokines in renal of SHR rats. And also, PA 45 mg/kg showed the best effect.

### PA decreased the level of cellular inflammatory factors and oxidative damage factor in renal of SHR rats

The results showed that, the expression of NOX4 increased significantly (Fig. 3b, d), indicated that there was a certain degree of oxidative damage. As shown in Table 3, the content of COX2 and MDA in renal of SHR were up-regulated significantly (*P* < 0.01), indicating the oxidative damage aggravating. Also, the content of IL-1, ET-1, E-selectin and MCP-1 in renal of SHR were increased significantly (*P* < 0.01), indicating the inflammatory response occurrence. After treatment with PA, the levels of above indicators in each group were significantly reduced (*P* < 0.01 or *P* < 0.05), indicated that PA could relieved the oxidative damage and inflammatory response in SHR rats. Among the different dose, 45 mg/kg PA showed the optimal effect.

**Table 3:** Influence of PA on inflammatory factors and oxidative damage factor in renal of SHR rats

Groups	COX2 (ng/mL)	IL-1 (pg/mL)	ET-1 (pg/mL)	MDA (nmol/mL)	E-selectin (ng/mL)	MCP-1 (pg/mL)
WKY	378.43 ± 24.31	659.19 ± 34.05	2.15 ± 0.52	61.53 ± 2.11	34.90 ± 2.63	2.5 ± 0.49
SHR	685.76 ± 100.95^**^	1148.3 ± 222.08^**^	7.11 ± 1.17^**^	72.97 ± 3.33^**^	43.85 ± 1.37^**^	6.89 ± 1.01^**^
SHR + HCTZ	422.36 ± 63.29^##^	677.76 ± 61.24^##^	1.8 ± 0.51^##^	64.23 ± 3.63^#^	41.67 ± 0.54	3.85 ± 1.03^##^
SHR + 15	373.16 ± 11.49^##^	1142.4 ± 177.19	2.09 ± 0.58^##^	69.33 ± 6.1	42.99 ± 2.78	3.65 ± 0.96^##^
SHR + 30	336.36 ± 93.19^##^	646.93 ± 43.66^##^	1.81 ± 0.66^##^	66.71 ± 5.76	39.52 ± 0.75^#^	3.04 ± 0.87^##^
SHR + 45	205.28 ± 22.6^##^	468.16 ± 60.59^##^	2.46 ± 1.36^##^	64.54 ± 3.36^#^	38.48 ± 3.44^#^	2.88 ± 0.50^##^

Data are expressed as mean ± SD, *n* = 8 per group. ^*^ P < 0.05, ^**^ P < 0.01 vs. WKY group; ^#^* P* < 0.05, ^##^* P* < 0.01 vs. SHR group

### PA inhibited RAAS activationin renal of SHR rats

As shown in Table 4, the levels of ACE, REN, Ang II, ALD in renal of SHR were up-regulated significantly (*P* < 0.01), suggesting excessive activation of RAAS system. After treatment with PA, the levels of REN, ACE, Ang II, ALD in each group were significantly reduced (*P* < 0.01 or *P* < 0.05), indicated that PA could inhibited the overactivation of RAAS in SHR rats. Along with the increase of dose, the improvement of PA showed better.

**Table 4:** Influence of PA on RAAS in renal of SHR rats

Groups	REN (pg/mL)	ACE (pg/mL)	Ang II (pg/mL)	ALD (pg/mL)
WKY	2596.45 ± 236.86	51.36 ± 7.48	301.95 ± 73.77	1705.31 ± 15.65
SHR	3596.97 ± 620.26^**^	80.77 ± 9.24^**^	648.23 ± 38.12^**^	2765.99 ± 647.78^**^
SHR + HCTZ	2905.44 ± 426.38^##^	60.68 ± 4.70^#^	378.18 ± 123.14^##^	1645.66 ± 212.40^##^
SHR + 15	3774.94 ± 331.34	57.67 ± 12.33^#^	785.24 ± 48.99	1788.35 ± 417.88^##^
SHR + 30	3158.06 ± 188.74	51.80 ± 12.10^##^	624.22 ± 8.31	1581.62 ± 92.88^##^
SHR + 45	2807.34 ± 214.77^##^	47.07 ± 11.08^##^	309.38 ± 8.73^##^	1508.14 ± 90.46^##^

Data are expressed as mean ± SD, *n* = 8 per group. ^*^ P < 0.05, ^**^ P < 0.01 vs. WKY group; ^#^* P* < 0.05, ^##^* P* < 0.01 vs. SHR group

### PA down-regulated the expression of MAPK signaling pathway proteins in renal of SHR rats

**Fig. 5:**
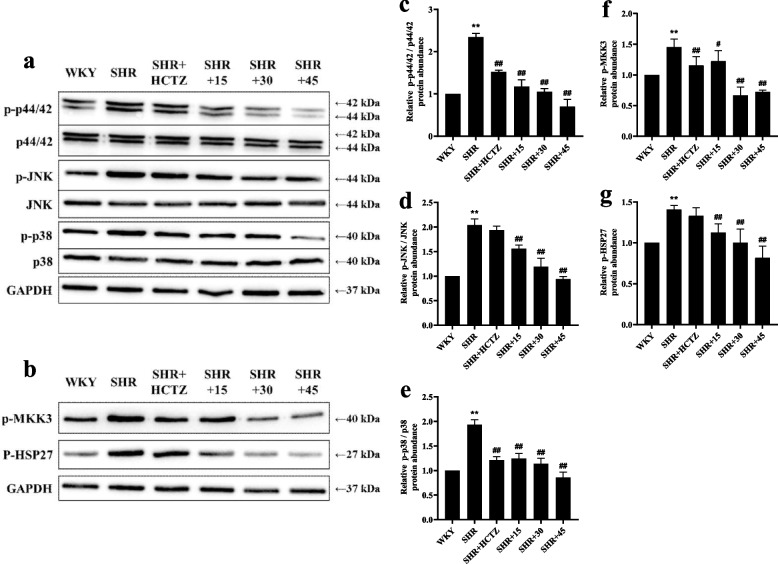
Effect of PA on the MAPK signalling pathway of renal in SHR rats. **a** The expression of p-p44/42 / p44/42, p-JNK / JNK, p-p38 / p38 using western blot. **b** The expression of p-MKK3 and p-HSP27 using western blot. **c**,** d**,** e** The relative protein abundance of p-p44/42 / p44/42, p-JNK / JNK, p-p38 / p38 by Image Lab analysis software. **f**,** g** The relative protein abundance of p-MKK3 and p-HSP27 by Image Lab analysis software. Three independent parallel experiments were performed for each protein to ensure the accuracy and reliability of the results. ^**^ P < 0.01 vs. WKY group; ^#^ *P* < 0.05 or ^##^ *P* < 0.01 vs. SHR group

In SHR group, the expression of p-p44/42, p-JNK, p-p38 MAPK increased significantly, indicating the MAPK signaling pathway was activated. After treatment with PA, the expression of above proteins in each group were significantly diminished (*P* < 0.01 or *P* < 0.05), indicated that PA restrainted the activation of MAPK signaling pathway in renal of SHR rats (Fig. 5a, c, d, e). Among them, PA also down-regulated the expression of p-MKK3, p-HSP27 (Fig. 5b, f, g), suggested that PA maybe showed the anti-renal fibrosis effect in SHR mainly through p38 MAPK pathway. 45 mg/kg PA showed the best effect.

### PA promoted the cell viability in the high NaCl-induced NRK52e cells

**Fig.6:**
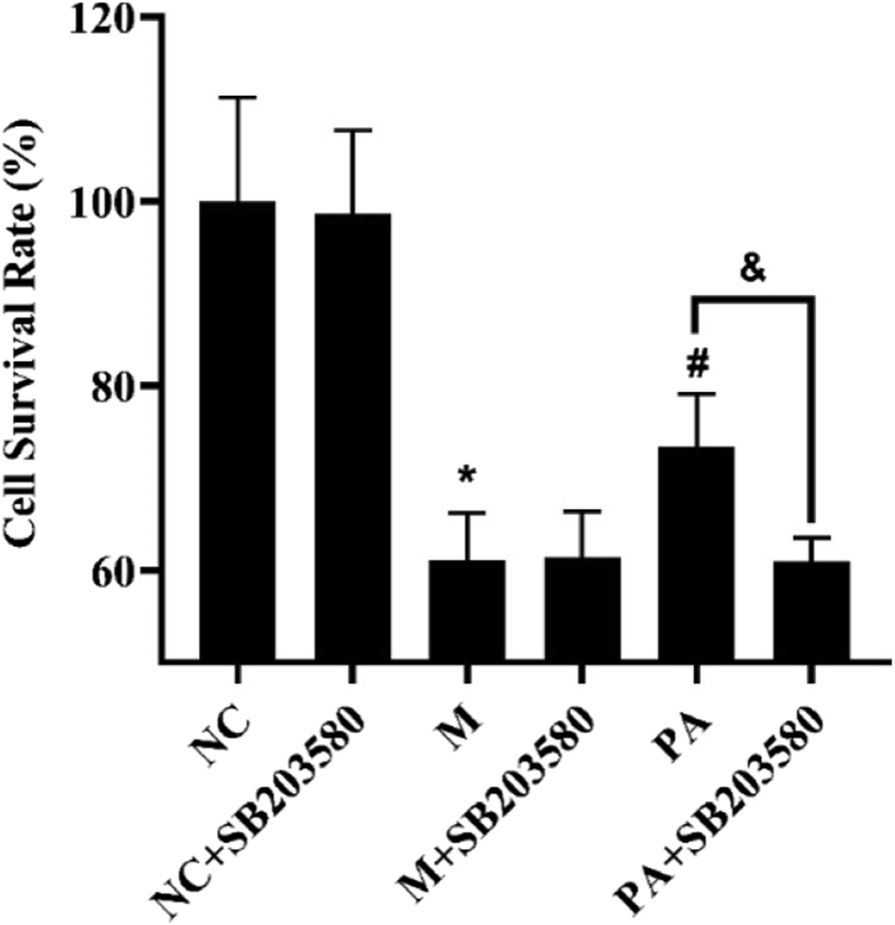
Effect of PA on the cell survival rate in the high NaCl-induced NRK52e cells. *n* = 6. ^*^ *P* < 0.05 vs. NC group; ^#^ *P* < 0.05 vs. M group; ^&^ *P* < 0.05 vs. PA group

Due to the significant improvement effect of PA on renal fibrosis in SHR rats and the remarkable regulation effect on p38 MAPK signaling pathway, we selected the high NaCl-induced NRK52e cells to further explore the potential mechanism of action of PA on anti-fibrosis. As the hypertonic situmulation, the cell viability rate of model group was decreased significantly, while PA could upregulated the cell viability rate, which could be blocked by SB203580 (p38 MAPK antagonists) (*P* < 0.01 or *P* < 0.05) (Fig. 6).

### SB203580 reversed the inhibition of PA on the fibrosis factor in the high NaCl-induced NRK52e cells

**Fig. 7:**
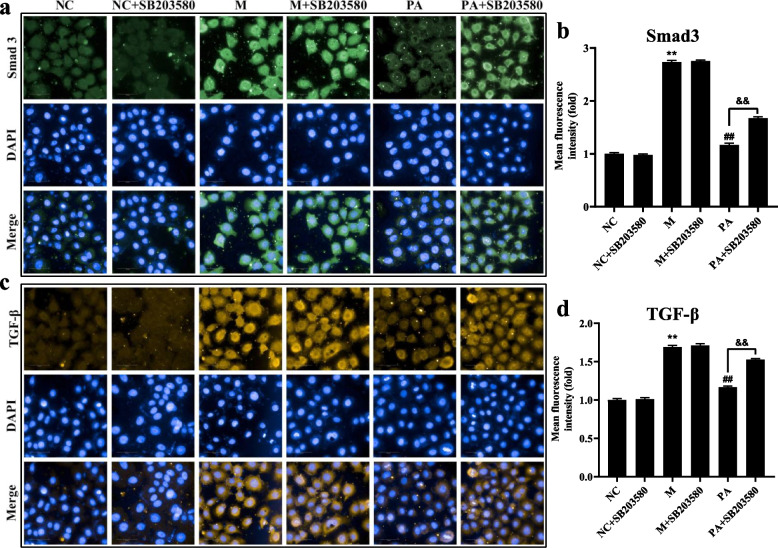
Effect of PA (with/without SB203580) on the fibrotic factor in the high NaCl-induced NRK52e cells. **a** DAPI and Smad3 double staining showed the degree of fibrosis in high NaCl-induced NRK52e cells using HCS. **b** The mean fluorescence intensity of Smad3 by Harmony 4.8. **c** DAPI and TGF-β double staining showed the degree of fibrosis in high NaCl-induced NRK52e cells using HCS. **d** The mean fluorescence intensity of TGF-β by Harmony 4.8. *n* = 6. ^**^ *P* < 0.01 vs. NC group; ^##^ *P* < 0.01 vs. M group; ^&&^ *P* < 0.01 vs. PA group

High NaCl-induced hypertonic stimulation resulted in the overexpression of Smad3 and TGF-β (*P* < 0.01), indicating that the hypertonic stimulation resulted in the fibrosis factor augmenting. Meanwhile, SB203580 remarkable restricted the amelioration of PA on the fibrosis factor in the high NaCl-induced NRK52e cells (Fig. 7).

### SB203580 restrainted the inhibition of PA on ROS production in the high NaCl-induced NRK52e cells

**Fig. 8:**
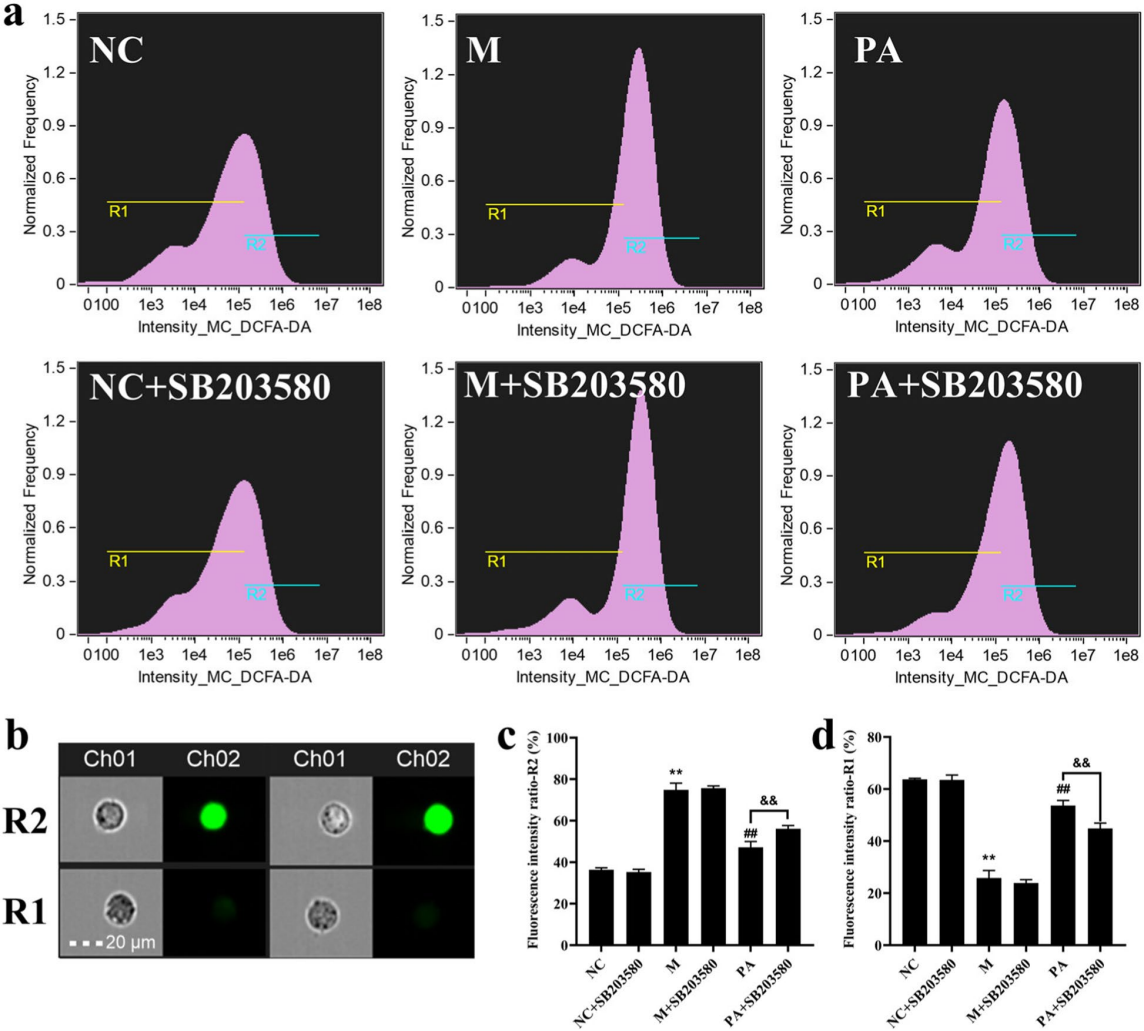
Effect of PA (with/without SB203580) on the RAAS and ROS in the high NaCl-induced NRK52e cells. **a** Fluorescence intensity with DCFH-DA in high NaCl-induced NRK52e cells using FCM. **b** Representative images of bright field (Ch01) and fluorescence channel (Ch02) with NRK52e cells by FCM. **c**,** d** The levels of ROS in NRK52e cells. *n* = 6. ^**^ *P* < 0.01 vs. NC group; ^##^ *P* < 0.01 vs. M group; ^&&^ *P* < 0.01 vs. PA group

As shown in Fig. 8, high NaCl-induced hypertonic stimulation led to ROS production and release (*P* < 0.01) (Fig. 8). PA intervention significantly diminished ROS level, but the improvement of PA was significantly antagonized by SB203580 (*P* < 0.01).

### SB203580 limited the inhibition of PA on the overactivation of RAAS and cytokines in the high NaCl-induced NRK52e cells

**Fig.9:**
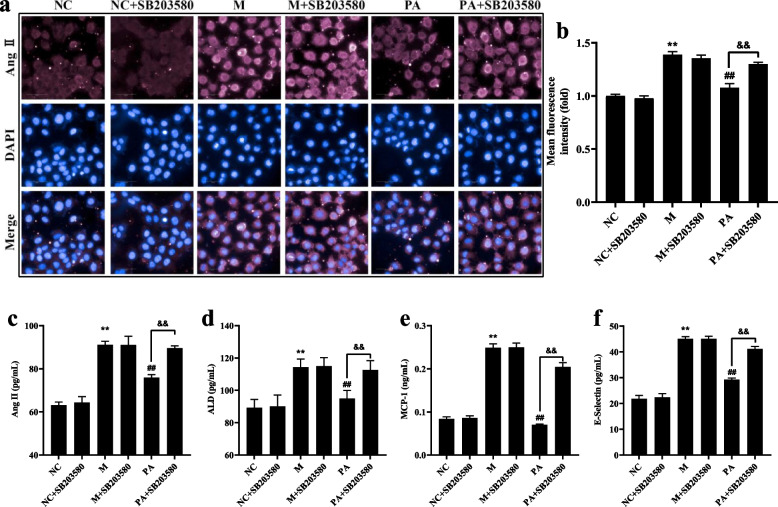
Effect of PA (with/without SB203580) on the RAAS and cytokines in the high NaCl-induced NRK52e cells. **a** DAPI and Ang II double staining in high NaCl-induced NRK52e cells using HCS. **b** The mean fluorescence intensity of Ang II by Harmony 4.8. **c** (Ang II), **d** (ALD), **e** (MCP-1), **d** (E-Selectin) The level of RAAS and cytokines in supernatant in NRK52e cells by ELISA. *n* = 6. ^**^ *P* < 0.01 vs. NC group; ^##^ *P* < 0.01 vs. M group; ^&&^ *P* < 0.01 vs. PA group

As shown in Fig. 9, high NaCl-induced hypertonic stimulation resulted in Ang II, ALD, MCP-1 and E-Selectin overexpression (*P* < 0.01), indicating that the hypertonic stimulation resulted in the RAAS activation and secretion of cytokines. Meanwhile, SB203580 remarkable restricted the amelioration of PA on RAAS and cytokines in the high NaCl-induced NRK52e cells.

### SB interfered with the regulation of PA on MAPK signaling pathway in the high NaCl-induced NRK52e cells

**Fig. 10:**
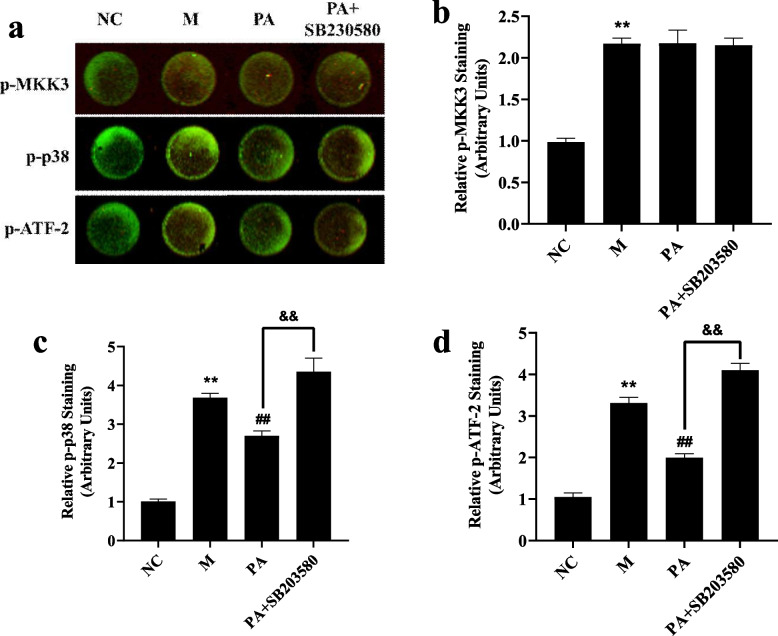
Effect of PA on the p38 MAPK signalling pathway in the high NaCl-induced NRK52e cells. **a** The fluorescence intensity of p-MKK3, p-p38, p-ATF-2 expression in high NaCl-induced NRK52e cells using In-Cell Western. **b** (p-MKK3), **c** (p-p38),** d** (p-ATF-2) The relative staining of p38 MAPK signalling pathway in NRK52e cells using Image Studio Ver 5.2 software. *n* = 6. ^**^ *P* < 0.01 vs. NC group; ^##^ *P* < 0.01 vs. M group; ^&&^ *P* < 0.01 vs. PA group

In high NaCl-induced NRK52e cells, the expression of phosphorylation with MKK3, p38 and ATF2 was increased significantly (*P* < 0.01). After administration of PA, the protein phosphorylation with p38 and ATF2 was significantly decreased (*P* < 0.01). While SB203580 antagonised the decrease the phosphorylation of p38 and its downstream protein ATF-2 by PA. However, it has no significant effect on the p38 upstream protein MKK3 (*P* < 0.01) (Fig. 10). It is suggested that the target of PA on the MAPK signalling pathway maybe is mainly on p38 MAPK.

### Interaction between PA and p38 MAPK active-site residues

**Fig. 11:**
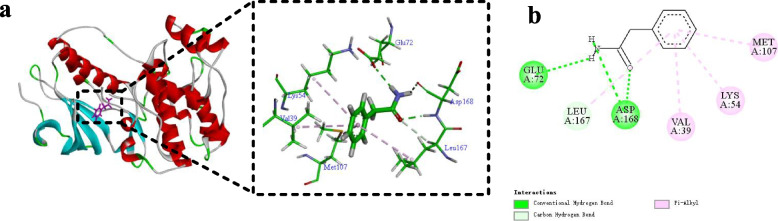
Three-dimensional (**a**) and two-dimensional (**b**) images of the molecular docking of PA and p38 MAPK crystal complex

To determine the binding position of PA and p38 MAPK, molecular docking technology was used for virtual docking (Fig. 11). PA interacts with multiple amino acid residues of p38 MAPK. The carbonyl oxygen atom forms a carbon-hydrogen bond with LEU167 and a conventional hydrogen bond with ASP168. The amino hydrogen atom forms a conventional hydrogen bond with GLU72 and ASP168. The Benzene ring forms a pi/alkyl hydrophobic region with LEU167, VAL39, LYS54 and MET107, which further enhances the binding ability of PA and p38 MAPK. The results indicate that PA has a certain binding capacity with p38 MAPK.

## Discussion

L.A has been recorded as having beneficial effects on water and swelling in the ancient literature of the past dynasties. In the previous material basis research, we found that PA is one of the largest components of L.A and showed a significant improvement on hypertension and estrogen-like activity [17, 19]. PA is a key pharmaceutical intermediate of atenolol and penicillin. Studies have shown that atenolol and dopamine, which have similar structure with PA, have beneficial effects on renal function and regulate renal fibrosis factor [16]. So, as the basic mother nucleus, PA may have a potential protective effect on renal injury. Meanwhile, PA has low molecular weight, is easy to prepare and obtain, and has a strong druggability. Renal fibrosis, as one of the important complications of hypertension, severely affects the survival rate and treatment effect of patients in its disease progression. Therefore, in addition to lowering blood pressure, the development of natural drugs that can effectively reverse the course of renal fibrosis has made a significant contribution to the prevention and treatment of hypertension. Thus, we consider whether the antihypertensive effect of PA is related to the improvement on renal fibrosis. Therefore, SHR model was used to explore the potential mechanism of PA on anti-renal fibrosis effect and a high NaCl-induced renal cell model was used to verify its possible mechanism.

Spontaneously hypertensive rats (SHR) is genetically determined by multiple genes, which is very similar to human hypertension. It is an ideal animal model for studying the pathogenesis of hypertension and screening antihypertensive drugs. Therefore, SHR rats were used in this study, Wistar-Kyoto rats (WKY) with the same background were used as blank control group. After treatment with PA, PA showed the effect of anti-hypertensive and promoting urination, also the morphological changes were alleviated and the inflammatory infiltration of renal was improved. Meanwhile, with the increase of dose, the improvement of PA showed better.

The existing theory holds that the specific pathogenesis of renal damage caused by hypertension mainly includes hemodynamic factors and non-hemodynamic factors, among which the non-hemorrhagic factors are mainly due to the activation of RAAS, resulting in an increase in serum levels of Ang II. The combined effects of oxidative stress and inflammation lead to the injury and remodeling of vascular endothelial cells in renal arterioles and induce the production of TGF-β1 in renal mesangial cells and tubular epithelial cells, and ultimately lead to renal fibrosis [21, 22]. Changes in renal structure can affect the normal physiological function of the renal. The results indicated that the renal structure was changed and the CVF was enhanced significantly in renal of SHR. PA improved the renal pathological structure and reduce the CVF.TGF-β1 is a key factor in renal fibrosis, which can activate the renal interstitial fibroblasts and induce the occurrence and development of renal interstitial fibrosis, and induce the expression of FN. FN acts as a bridge and skeleton in the process of renal fibrosis. Our results revealed that PA could significantly diminish the levels of E-Selectin, TGF-β1, FN and MCP-1, and promote the development of renal interstitial fibrosis in SHR rats.

A large number of studies have found that TGF-β induce the NOX4 expression in various cell types (especially the kidney), and that ROS from NOX4 sources mediate TGF-β fibrosis [23, 24]. The ROS produced by NOX4 can significantly induce the recruitment of macrophages, production of inflammatory cytokines and E-Selectin, and increase the expression of MCP-1 [25], thus further improving the level of oxidative stress and promoting the progression of renal interstitial fibrosis [26]. Meanwhile, NOX-dependent redox signaling can also feedback to regulate fibrosis-related signaling pathways, thus indirectly promoting the fibrosis process. As seen from the results, with the increase of TGF-β1, the expression of NOX4 in renal of SHR rats was augmented, which led to the increase of MDA content, suggesting the occurrence of oxidative stress. PA intervention can significantly reverse these changes and restore the oxidative-antioxidant homeostasis.

In recent years, researchers have found that the RAAS activation in patients with hypertension is the main cause of renal injury [27]. Angiotensin II can increase blood pressure by stimulating the adrenocortical globular zone, promoting aldosterone secretion, water and sodium retention, stimulating sympathetic ganglions to raise norepinephrine secretion, increasing sympathetic neurotransmitters and the activity of specific receptors [28]. Excessive secretion of Ang II and ALD can promote vasoconstriction and reabsorption of water and sodium, aggravating hypertension [29]. As the main product of the RAAS system, Ang II can induce the activation of inflammatory and lead to the release of inflammatory cytokines [30]. Inflammation exerts a vital role in the development and complications of hypertension [31]. Our results showed that PA can significantly inhibit the overactivation of the RAAS system, thus improving the renal fibrosis. Meanwhile, PA could significantly decrease the levels of COX-2, IL-1 and ET-1, improve the inflammation in renal of SHR rats.

The MAPK family is an important part of the intracellular signal protein network and a common pathway through which various extracellular signals are transmitted to the nucleus [32]. In hypertension serum Ang II binds to its receptor AT1R to activate the intracellular MAPK signalling pathway due to the activation of RAAS. The results demonstrated that PA can restraint the phosphorylation of ERK1/2, JNK, especially p38 in renal of SHR rats.

P38 MAPK is involved in the signalling cascade that controls cellular responses to cytokines and stress [33–36]. MKK3 activate p38 MAPK by phosphorylation at Thr180 and Tyr182. Activated p38 MAPK has been shown to phosphorylate and activate MAPKAP kinase 2 [35] and then phosphorylate ATF-2 [37]. SB203580 is a selective inhibitor of p38 MAPK [37, 38]. The current studies have shown that the high NaCl-induced NRK52e cells can be further studied as an in vitro model of hypertensive induced renal fibrosis [18]. In order to elucidate the potential mechanism of PA against renal fibrosis in SHR rats, we used the high NaCl-induced NRK52e cells to observe the effect of combined or non-combined PA with SB203580 on renal fibrosis. The results showed that in the high NaCl-induced NRK52e cells, the improvement of PA on cell survival rate, fibrosis, ROS, RAAS and p38 MAPK signalling pathway was all blocked by SB203580, suggesting that PA does play a role via p38 MAPK through MAPK signalling pathway.

Molecular docking is a theoretical simulation method to predict binding patterns and affinity based on the properties of receptor and the interaction between receptor and drug molecules. In recent years, molecular docking has become an important technology in the field of computer-aided medicine research. In order to further clarify the interaction between PA and p38 and its potential binding position, we adopted the molecular docking technology of Discovery Studio 2020 Client. Through molecular docking analysis, it was found that the hydrophobic cavity of p38 MAPK occupy PA form strong hydrophobic interactions (such as carbon-hydrogen bond, conventional hydrogen bond, pi/alkyl hydrophobic region) with the active site residues (LEU167, ASP168, GLU72, VAL39, LYS54, MET107).

## Conclusions

PA Separated from *Lepidium apetalum* Willd. inhibited renal fibrosis via MAPK signalling pathway mediated RAAS and oxidative stress in SHR Rats.

## Supplementary Information

**Additional file 1.**

## Data Availability

All of the data used to support the findings of this study are available from the corresponding or the first authors upon reasonable request.

## Abbreviations

ACE: Angiotensin-converting enzyme
ALD: Adosterone
Ang II: Angiotensin II
AT1R: Angiotensin II Type 1 Receptor
BP: Blood pressure
BSA: Bovine serum albumin
COX2: Cyclooxygenase-2
CVF: Collagen volume fraction
DBP: Diastolic blood pressure
ECM: Extracellular matrix
ELISA: Enzyme-linked immunosorbent assay
ERK1/2: Extracellular signal-regulated kinase 1/2
ET-1: Endothelin-1
FCM: Flow cytometry
FN: Fibronectin
HCS: High content screening
HCTZ: Hydrochlorothiazide
HE: Hematoxylin–eosin
HPLC: High-performance liquid chromatography
IHC: Immunohistochemistry
IL-1: Interleukin-1
JNK: C-Jun N-terminal kinase
MAP: Mean arterial pressure
MCP-1: Monocyte chemotactic protein-1
MDA: Malondialdehyde
NF-κB: Nuclear factor-κB
NMR: Nuclear magnetic resonance
PA: 2-Phenylacetamide
PGE2: Prostaglandin E2
PRA: Plasma renin activity
PVDF: Polyvinylidene difluoride
RAAS: Renin-angiotensin aldosterone system
REN: Renin
ROS: Reactive oxygen species
SBP: Systolic blood pressure
SDS-PAGE: Sodium dodecyl sulphate–polyacrylamide gel electrophoresis
SHR: Spontaneously hypertensive rat
SOD: Superoxide dismutase
TBA: Thiobarbituric acid
TGF-β: Transforming growth factor-β
