# Supplementary material for: 2-phenylacetamide Separated from the seed of Lepidium apetalum Willd. inhibited renal fibrosis via MAPK pathway mediated RAAS and oxidative stress in SHR Rats
Source: BMC Complement Med Ther. 2023 Jun 23;23:207. doi: 10.1186/s12906-023-04012-w (PMC10290354; doi:10.1186/s12906-023-04012-w)

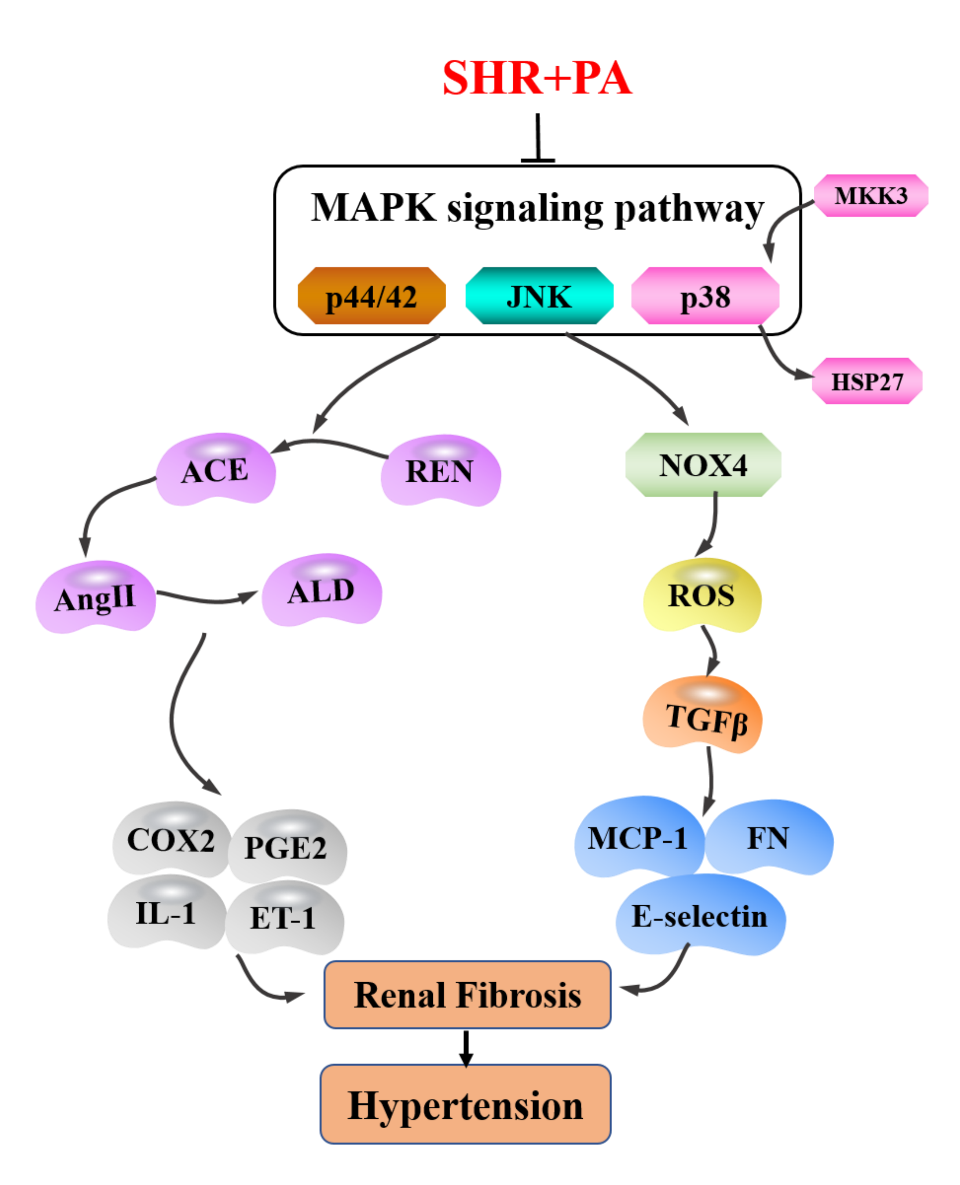


Fig. S1 PA Separated from Lepidium apetalum Willd. inhibited renal fibrosis via MAPK signalling pathway mediated RAAS and oxidative stress in SHR Rats.

**Fig. 5 a, b supplementary materials:**

**From left to right: WKY, SHR, SHR+HCTZ, SHR+15, SHR+30, SHR+45.**

**Three independent parallel experiments were performed for each protein.**

**alidation for p-p44/42：**


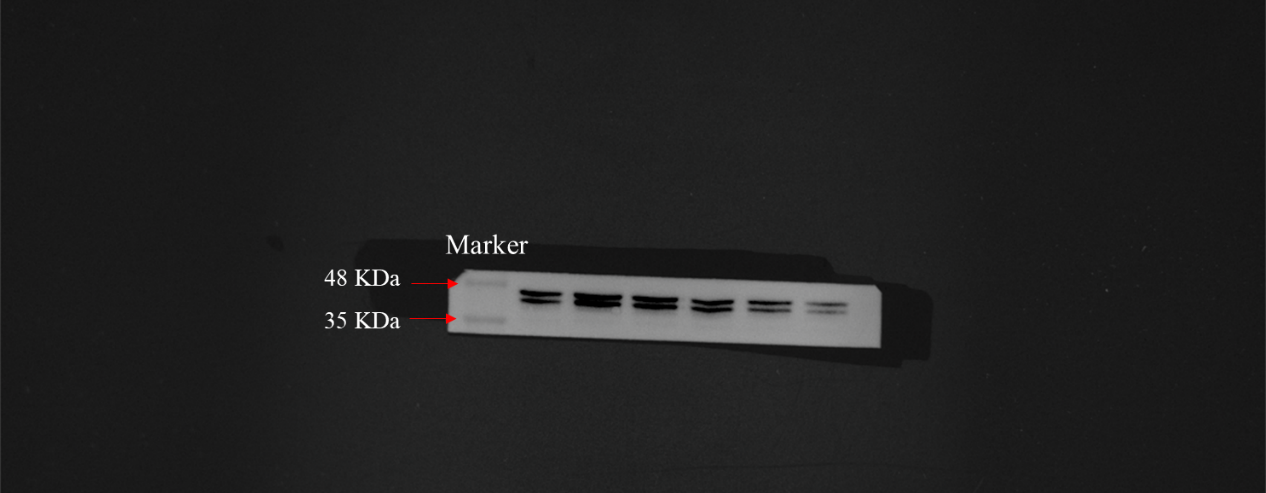


**
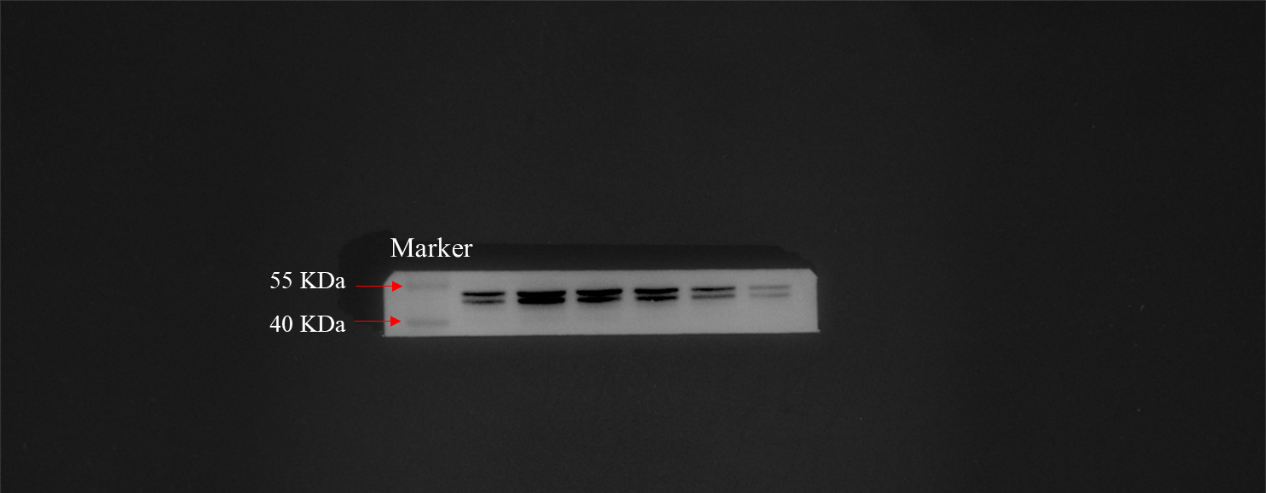
**

**
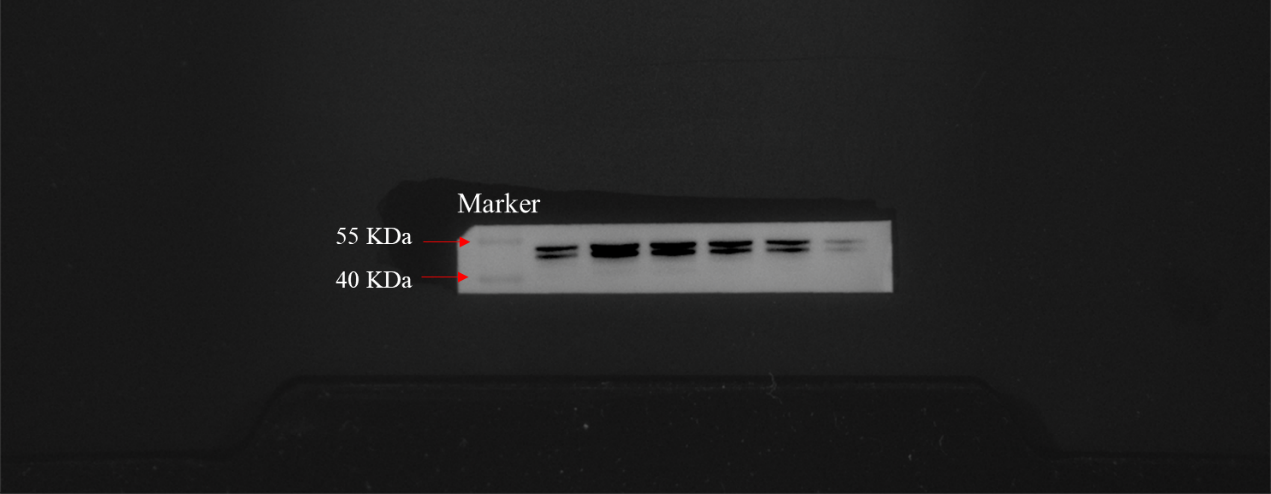
**

**alidation for p44/42**


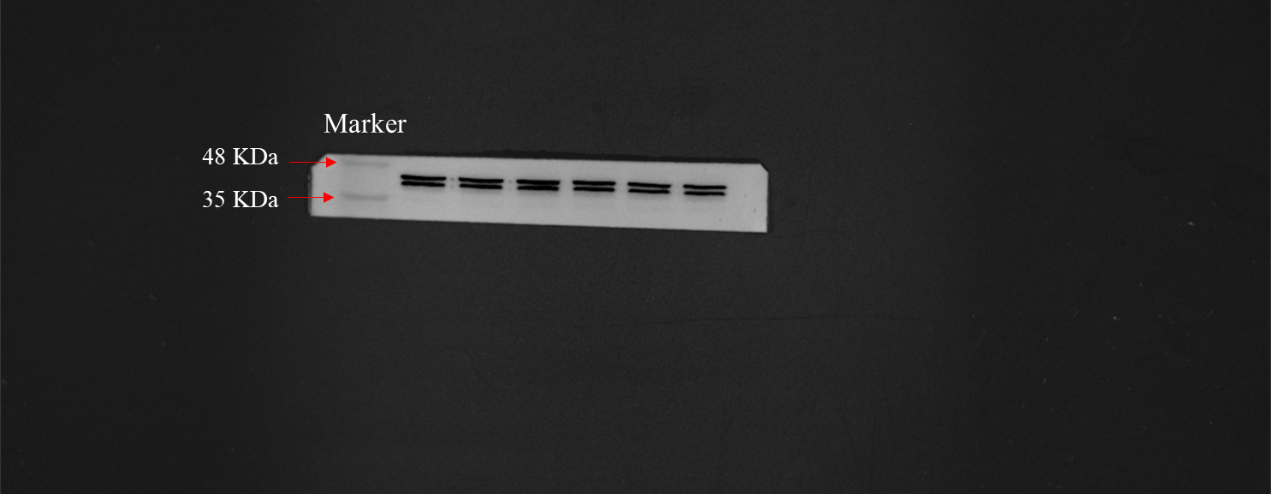


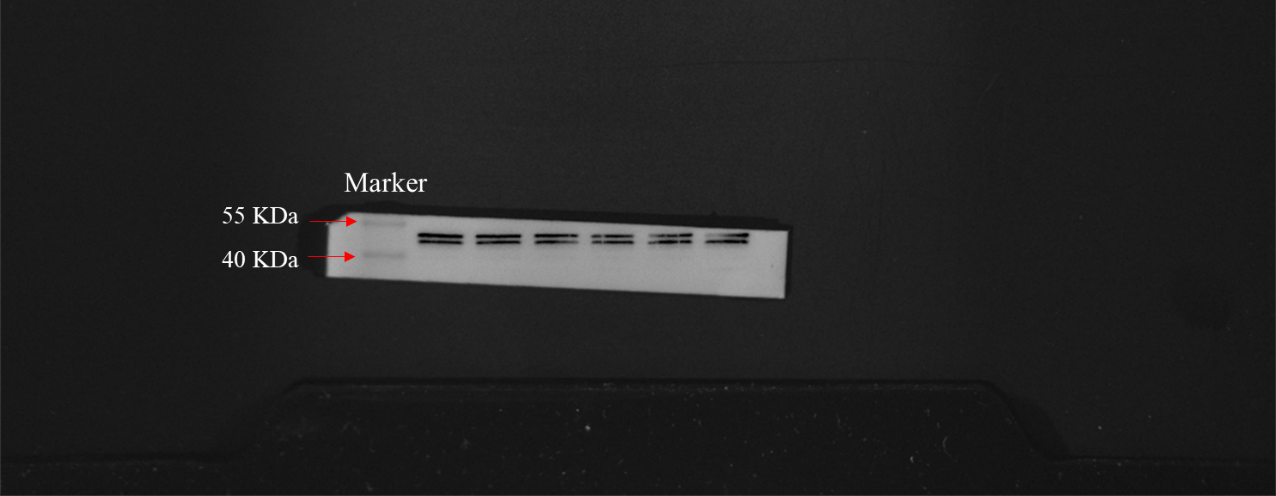


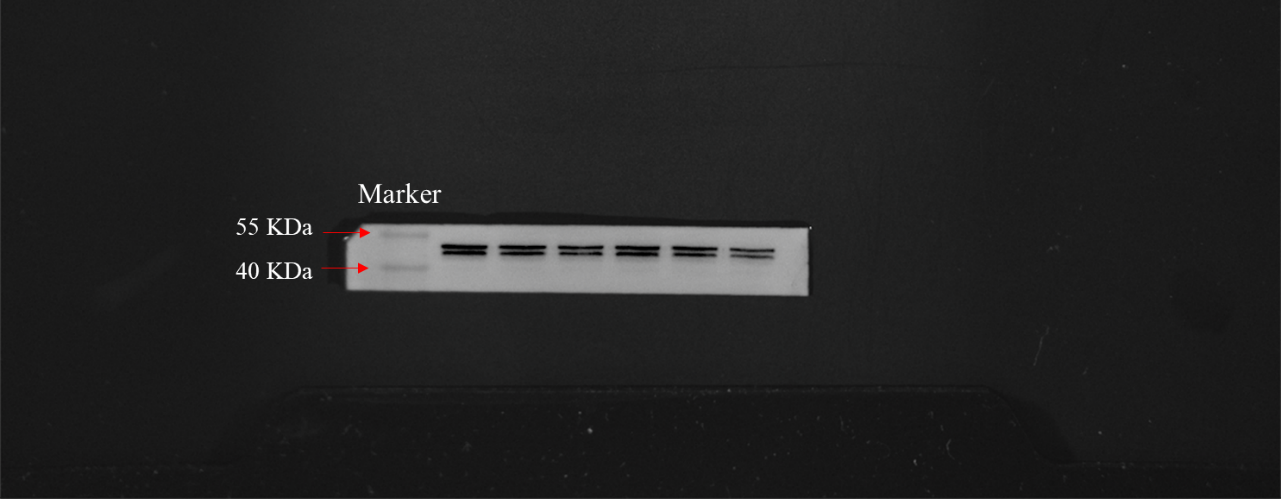


**alidation for p-JNK**


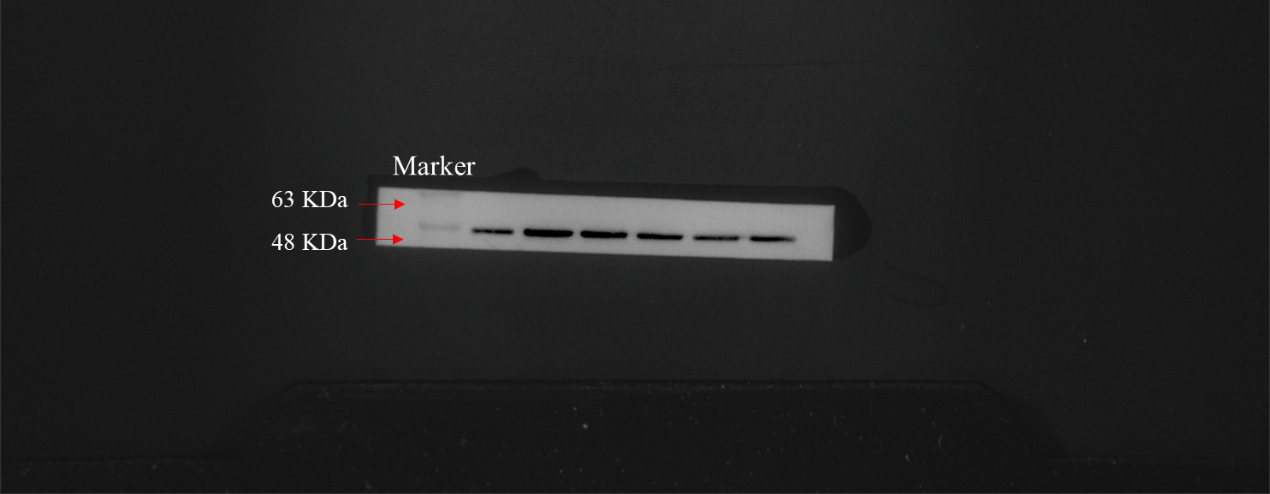


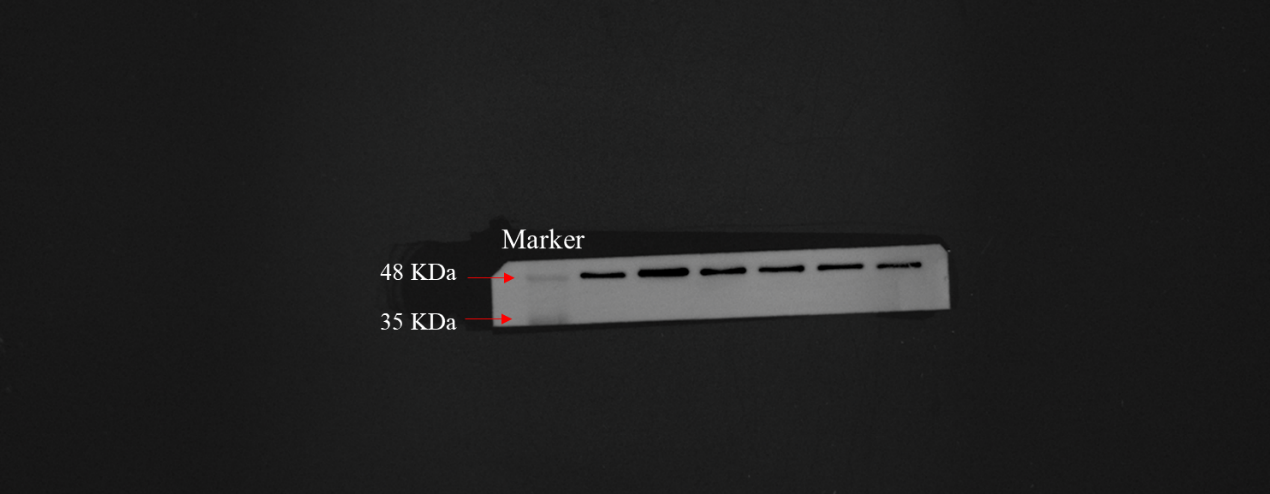


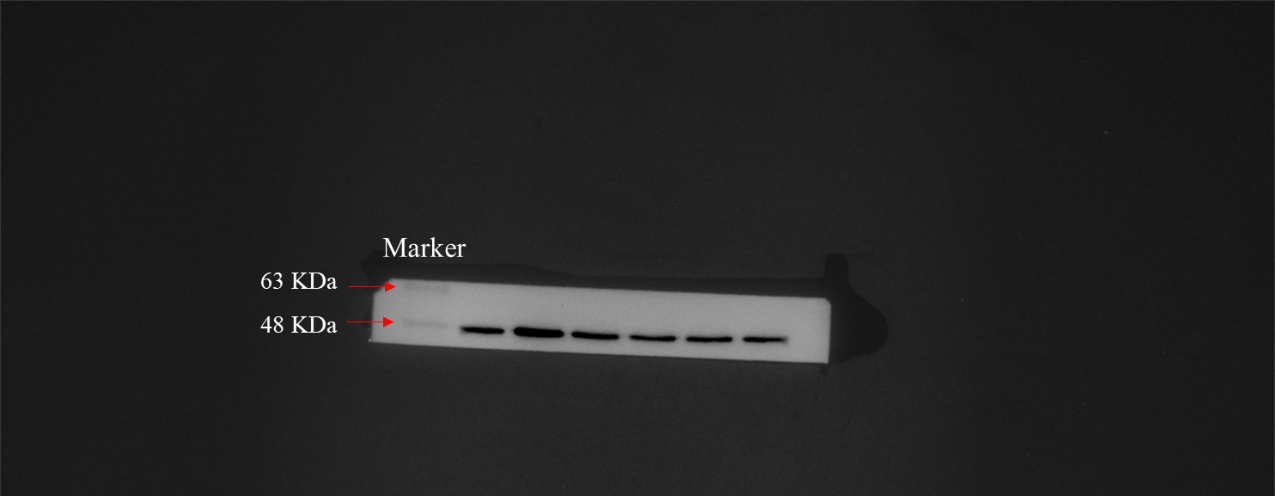


**alidation for JNK**


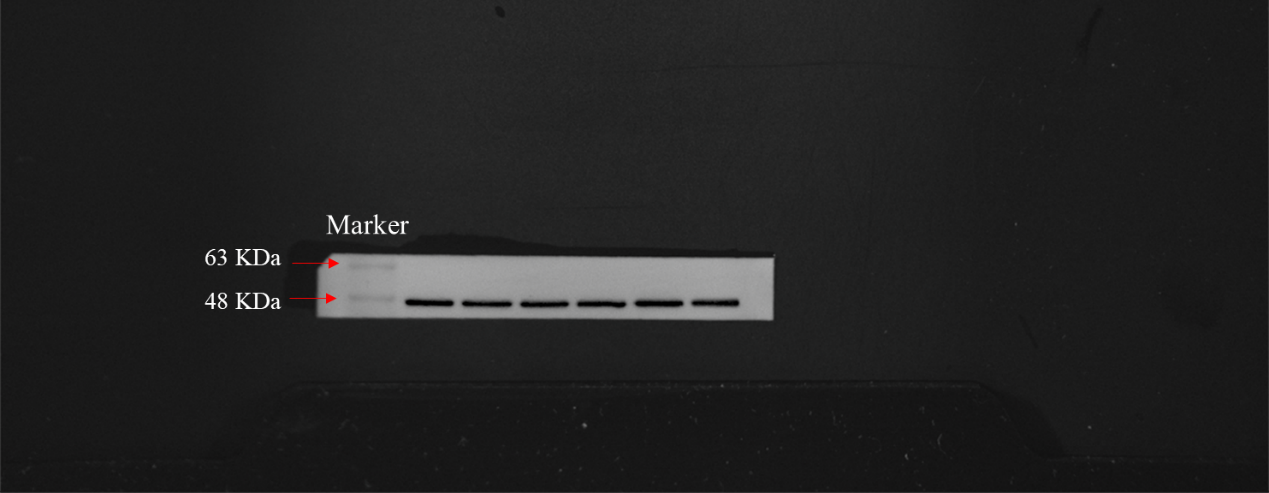


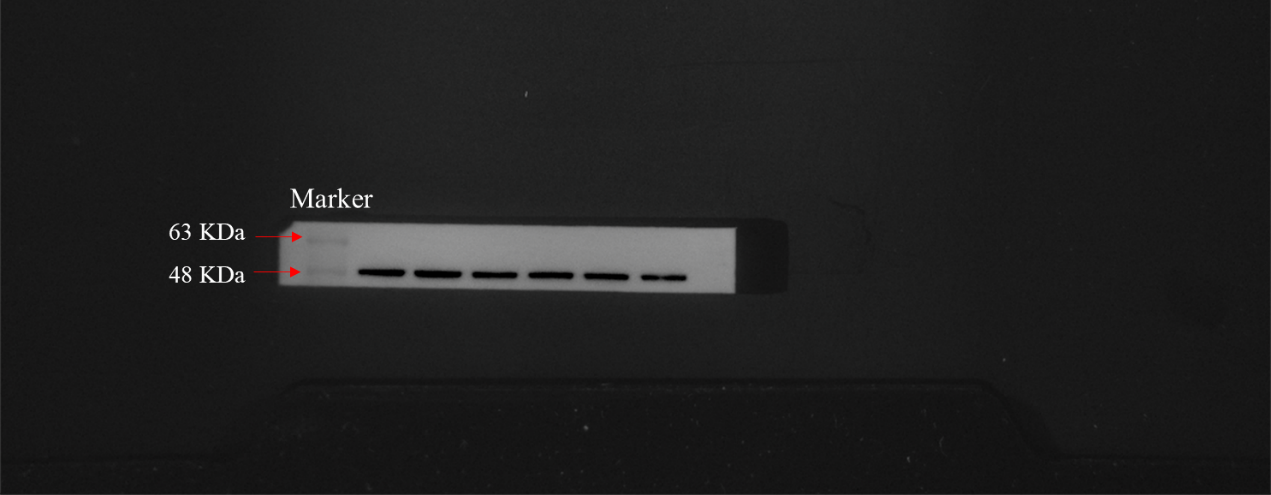


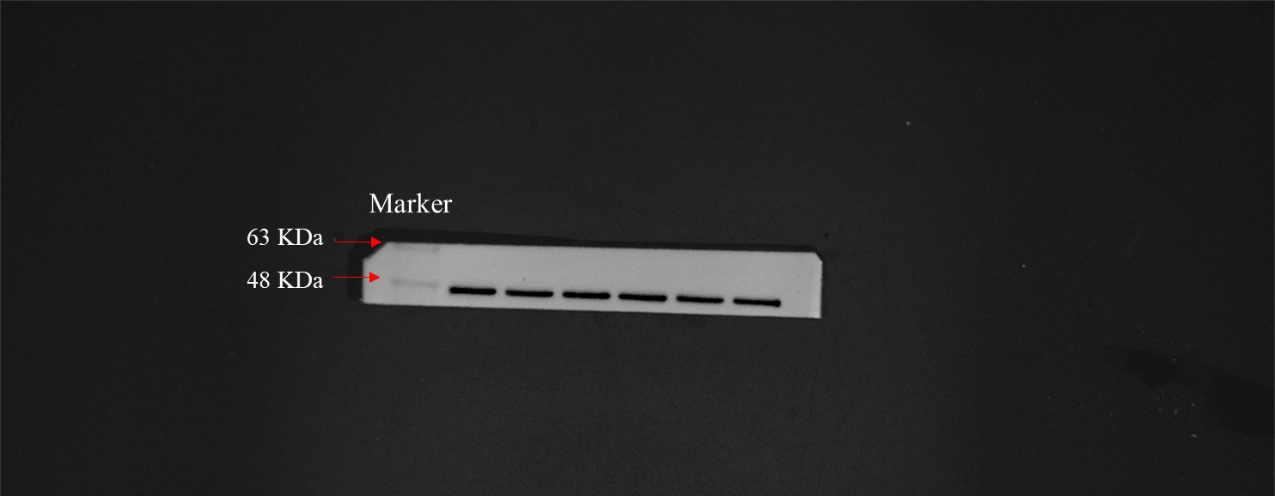


**alidation for p-p38**

**
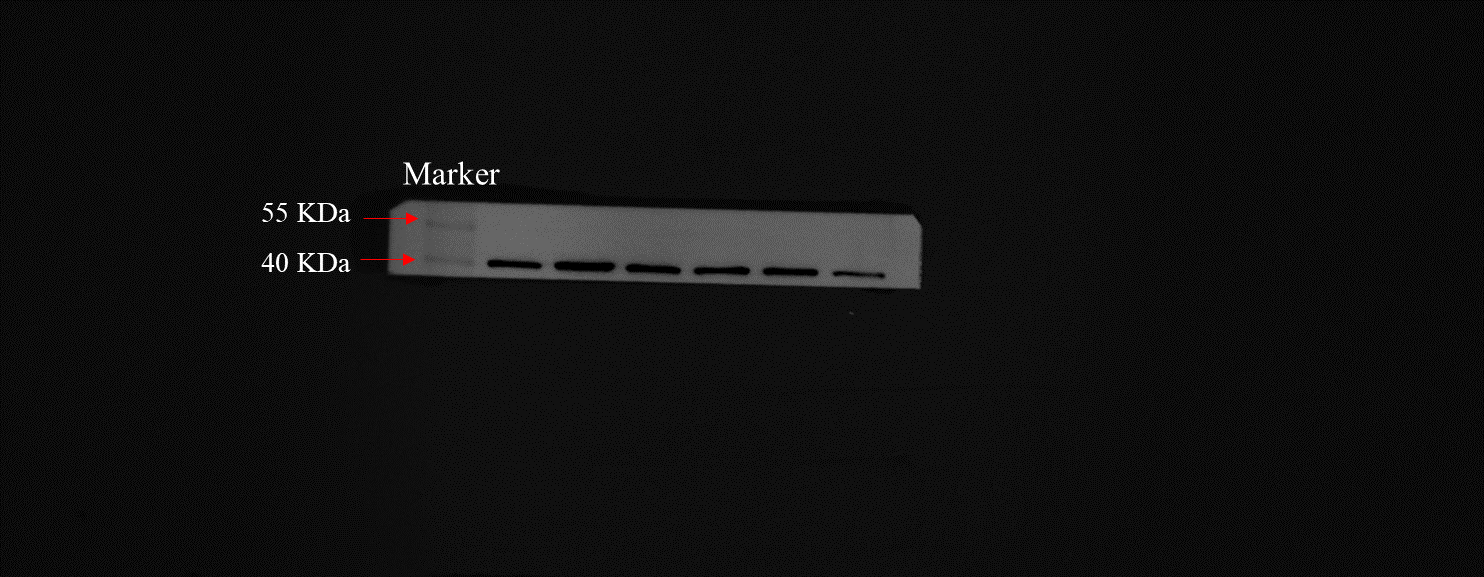
**

**
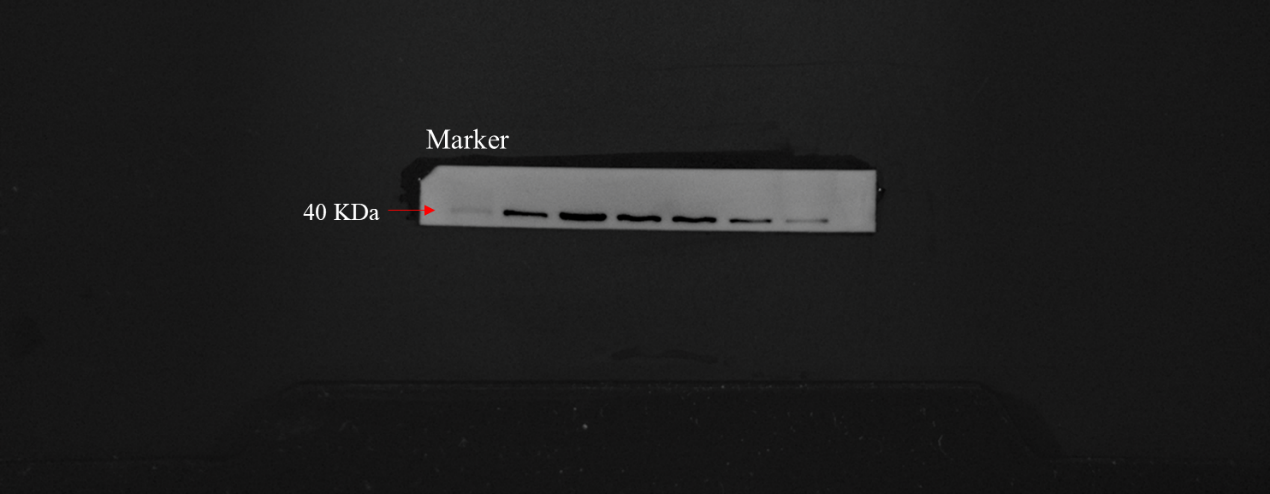
**

**
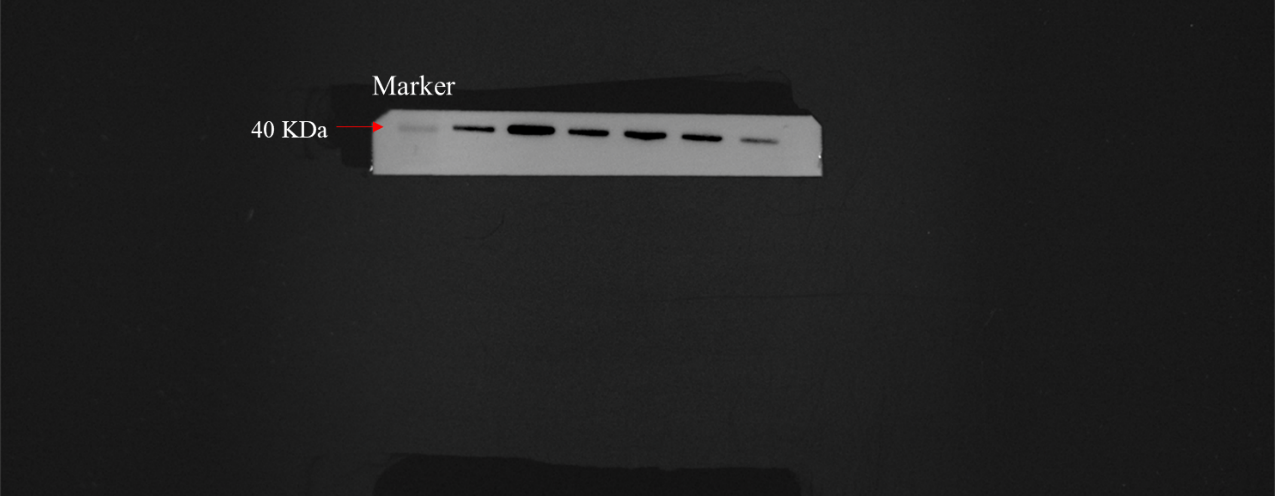
**

**alidation for p38**

**
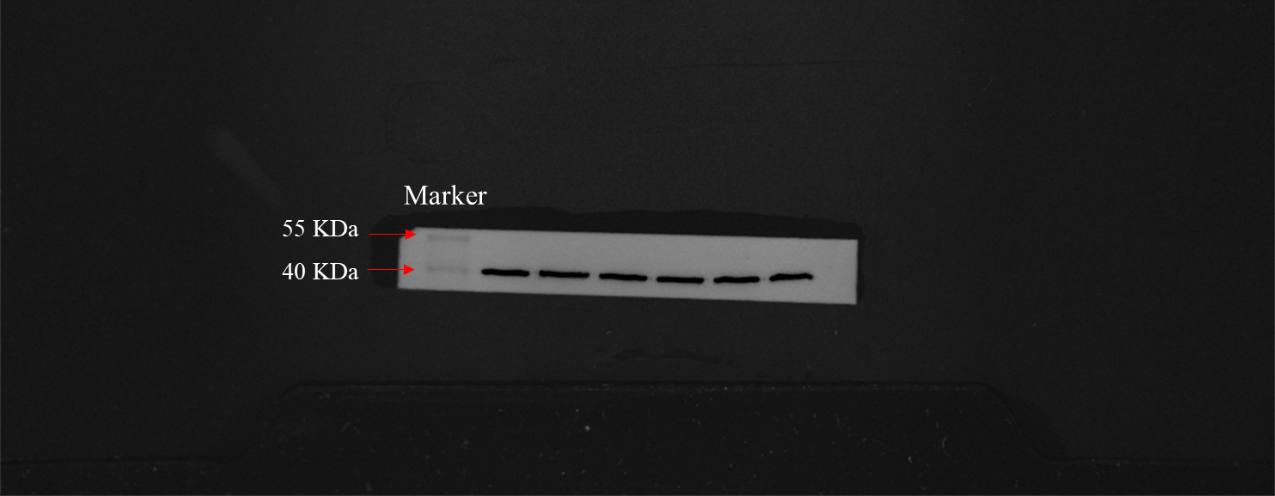
**

**
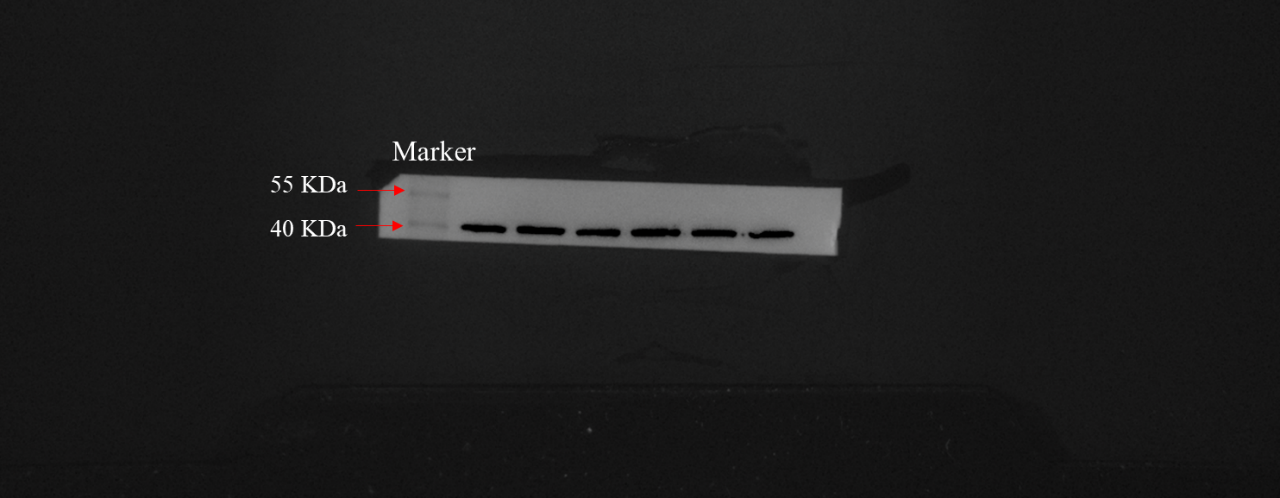
**

**
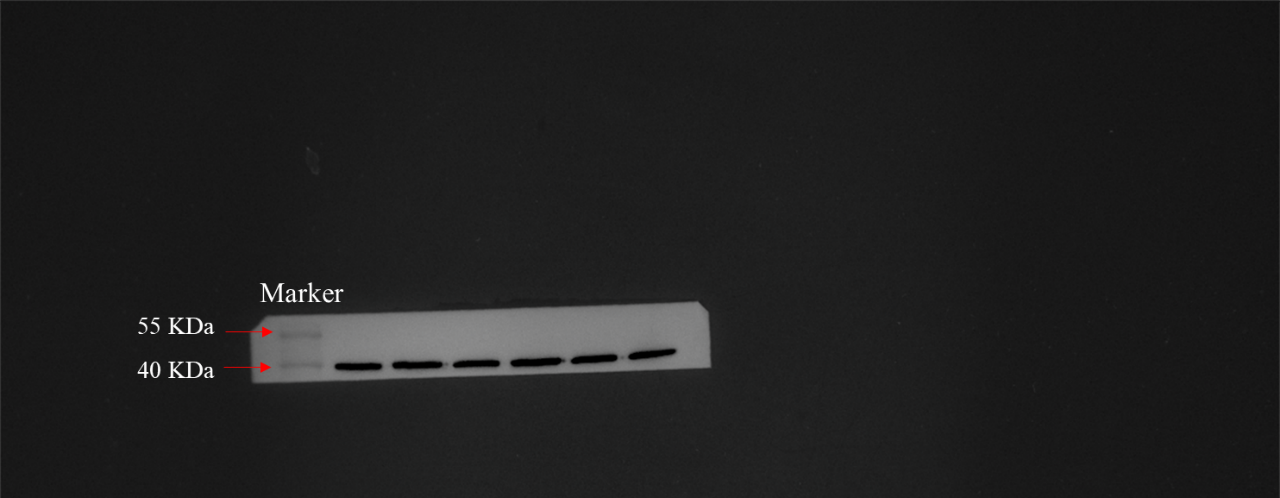
**

**alidation for p-MKK3**

**
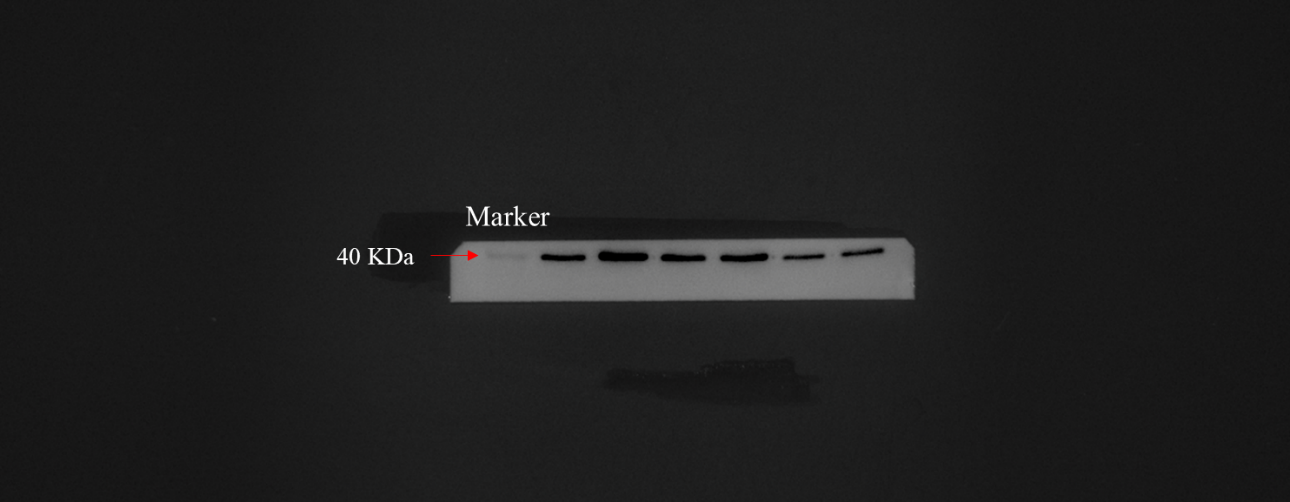
**

**
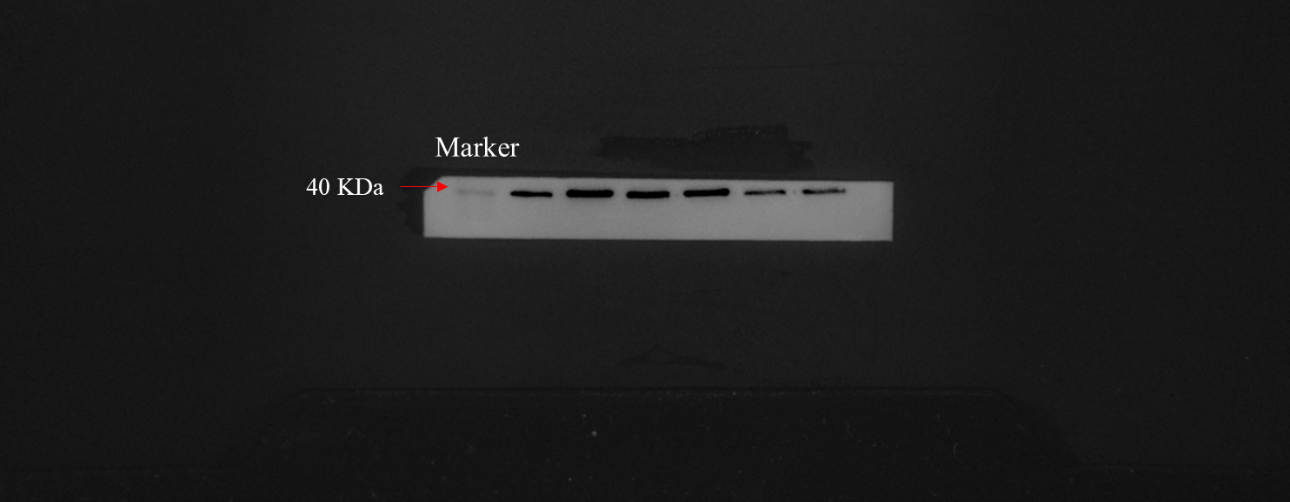
**

**
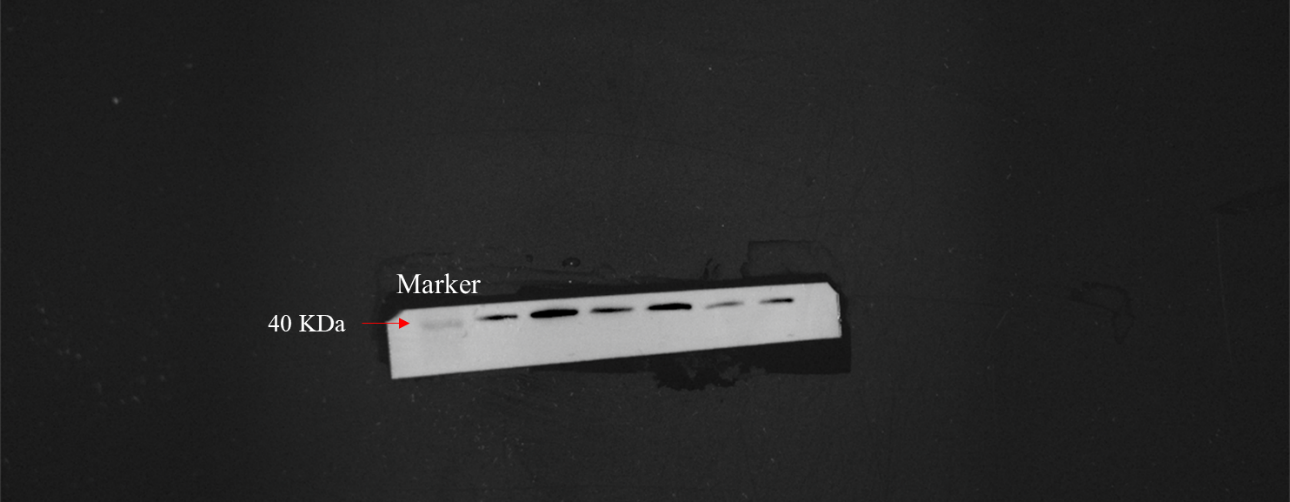
**

**alidation for p-HSP27**

**
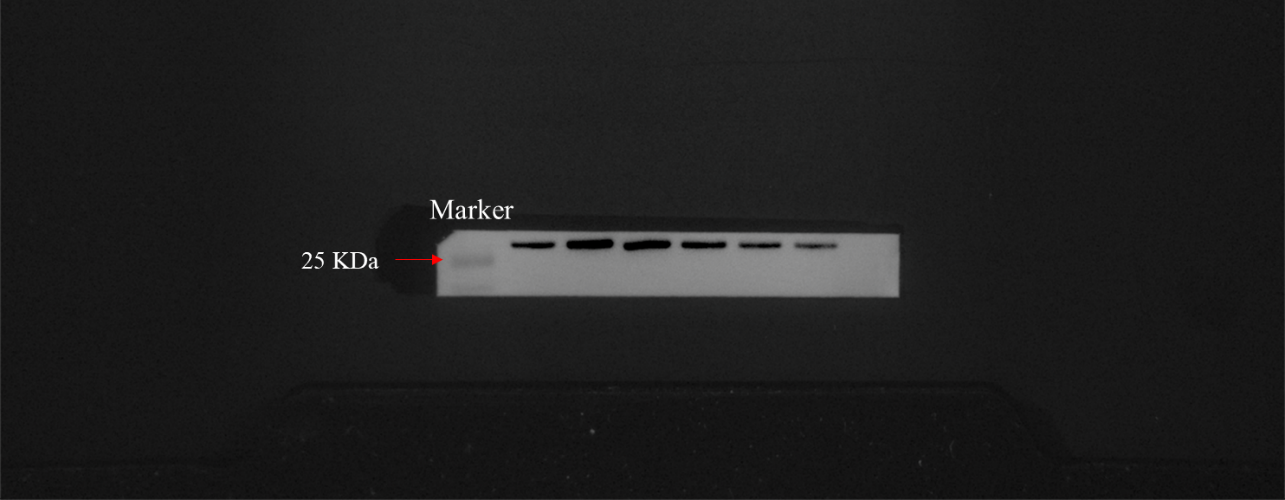
**

**
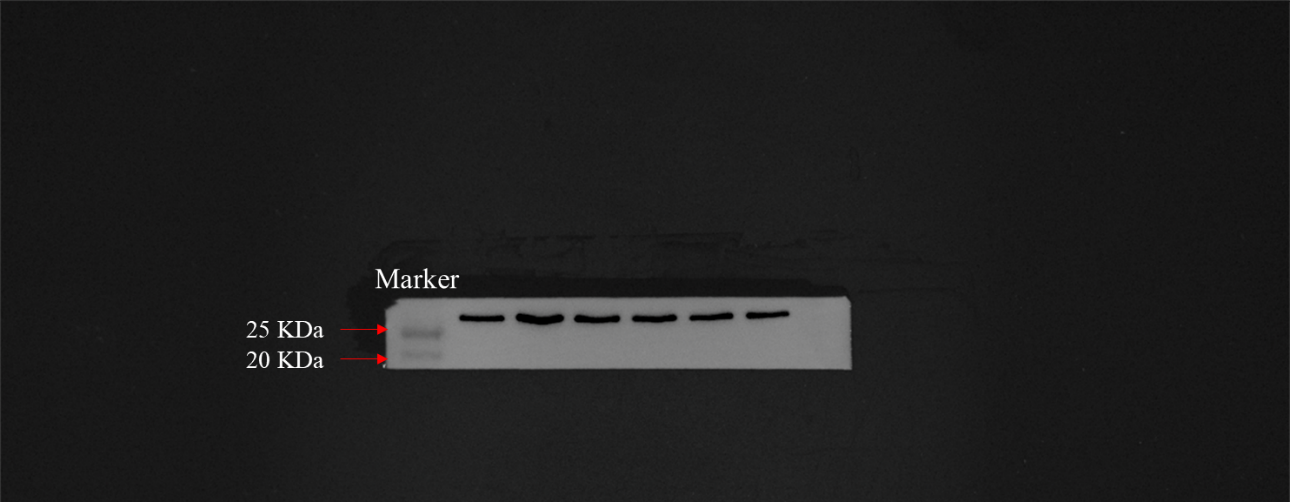
**

**
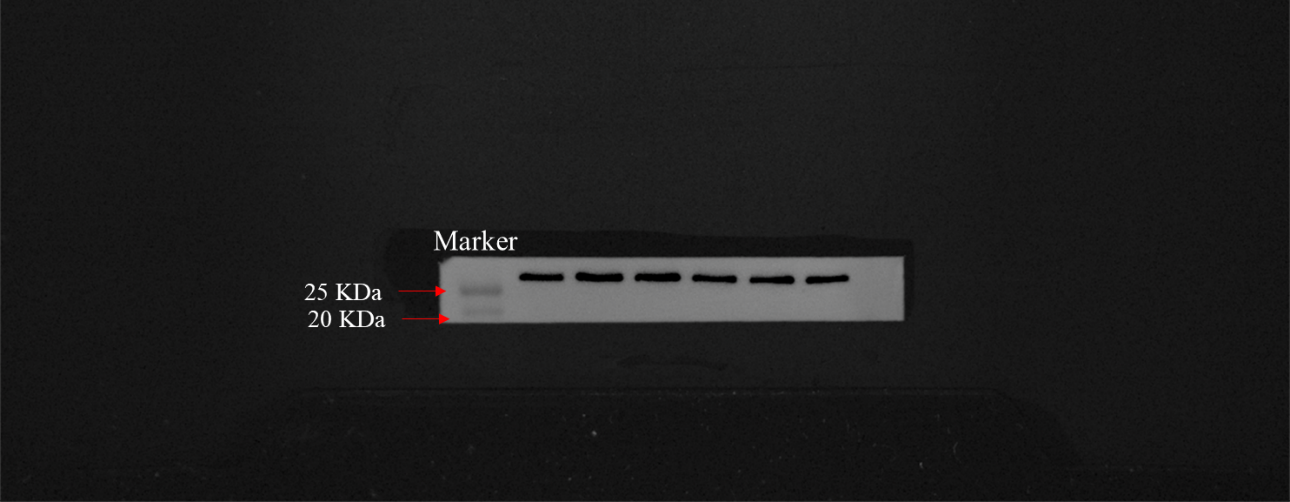
**

**alidation for GAPDH**

**
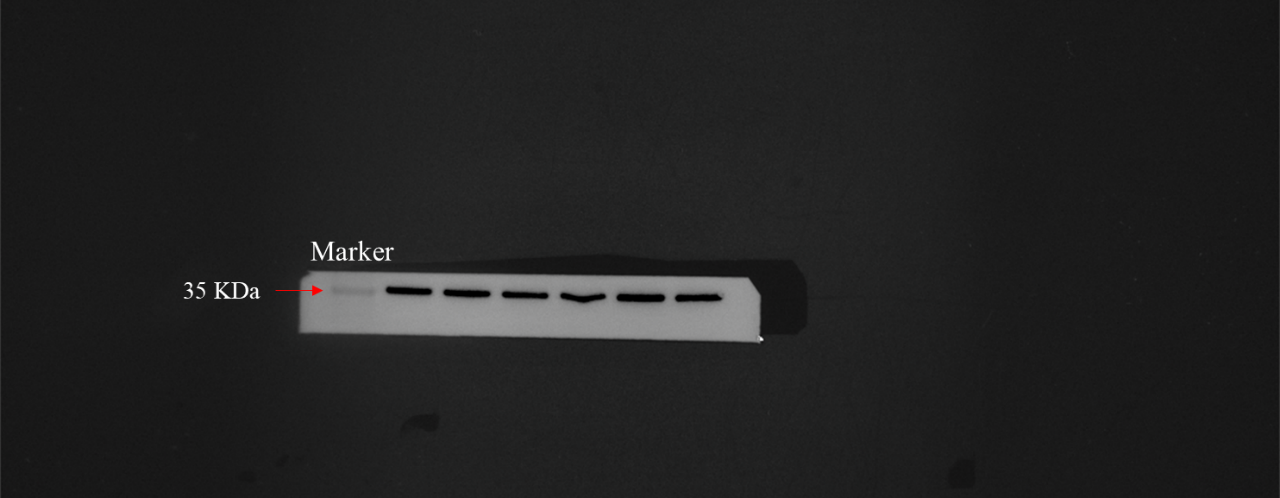
**


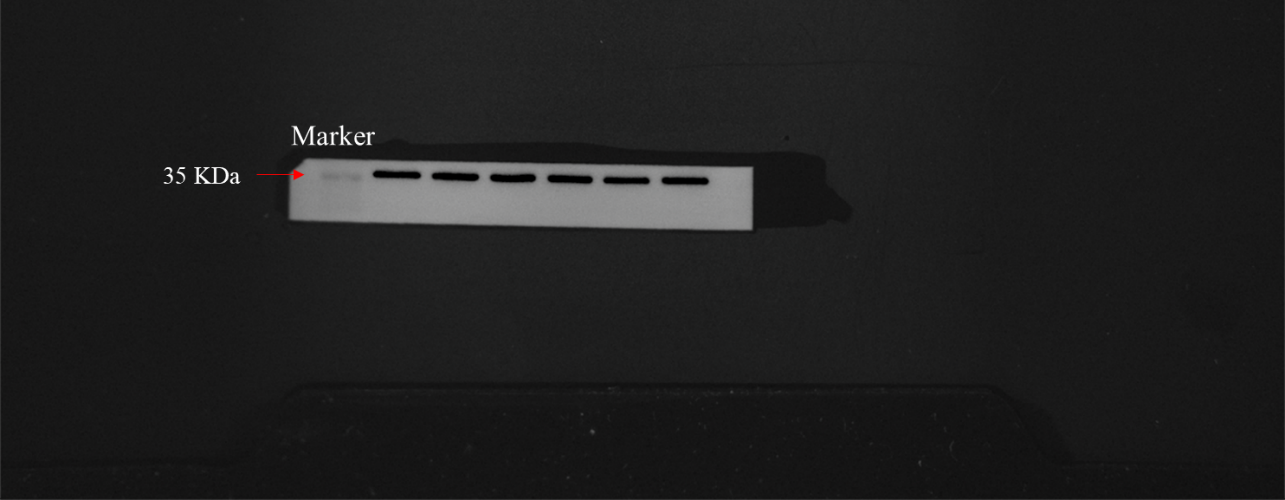


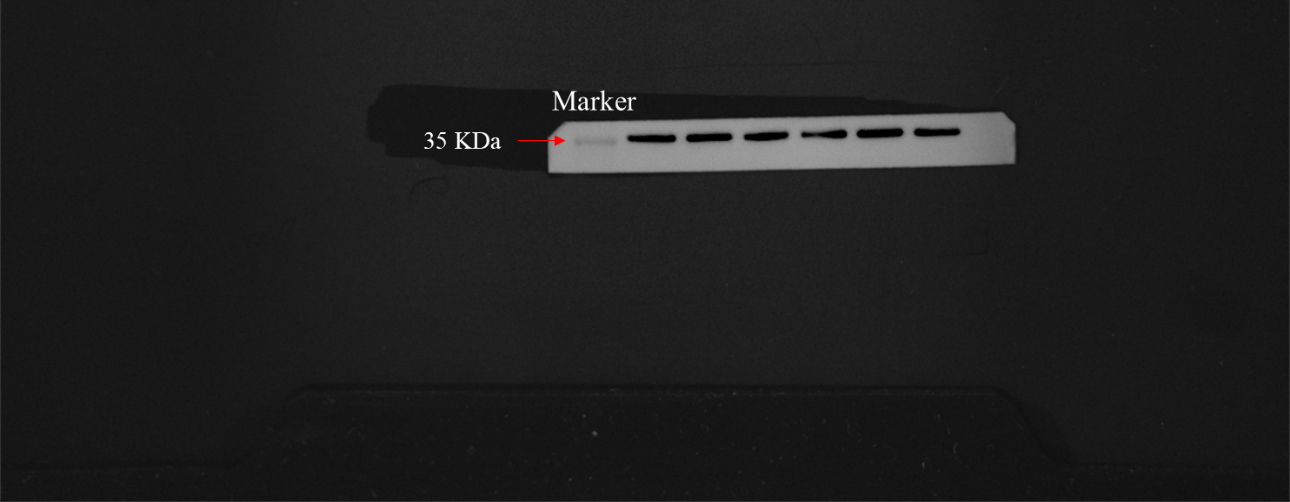

Supplement: Supplementary file 1 — Additional file 1. [file 12906_2023_4012_MOESM1_ESM.docx]
